# The four voltage-sensing domains of T-type calcium channels activate near the resting membrane potential

**DOI:** 10.1038/s41467-026-73077-1

**Published:** 2026-05-16

**Authors:** Marina Angelini, Moira McVicar, Savana Maxfield, Milosz Sokolowski, Sanjana Narang, Kyle Scranton, S. Suheda Yasarbas, Nicoletta Savalli, Scott A. John, Andreas Schwingshackl, Alan Neely, Antonios Pantazis, Michela Ottolia, Riccardo Olcese

**Affiliations:** 1 Division of Molecular Medicine, Department of Anesthesiology & Perioperative Medicine, David Geffen School of Medicine, University of California, Los Angeles, Los Angeles, CA USA; 2 Division of Cell and Neurobiology, Department of Biomedical and Clinical Sciences, Linköping University, Linköping, Sweden; 3 Department of Medicine, Division of Cardiology, David Geffen School of Medicine, University of California, Los Angeles, Los Angeles, CA USA; 4 Departments of Pediatrics, David Geffen School of Medicine, University of California, Los Angeles, Los Angeles, CA USA; 5 Centro Interdisciplinario de Neurociencias de Valparaíso, Facultad de Ciencias, Universidad de Valparaíso, Valparaíso, Chile; 6 Wallenberg Center for Molecular Medicine, Linköping University, Linköping, Sweden; 7 Department of Physiology, David Geffen School of Medicine, University of California, Los Angeles, Los Angeles, CA USA

**Keywords:** Ion channels in the nervous system, Ion transport, Calcium channels, Structural biology

## Abstract

Low-voltage-activated (LVA, T-type, or Ca_V_3), calcium-selective channels open in response to modest depolarizations, just above the resting membrane potential, supporting neuronal burst-firing patterns and spontaneous firing in cardiac pacemaker cells. How LVA-channels open at low voltages is unclear: traditional gating-current experiments suggest that LVA-channel voltage-sensing domains (VSDs) paradoxically require stronger depolarization to activate than pore opening. Using voltage-clamp fluorometry, we find that the activation of all four VSDs in human Ca_V_3.1-channels precedes opening in voltage, solving the longstanding conundrum. We also uncover confounding effects of La^3+^ (used for gating-current measurements) on VSD function and clarify the role of distinct LVA-channel structure S6^Cyto^. Ca_V_3.1-VSDs operate within a narrow voltage-range, resembling the VSDs of related Na_V_-channels more than those of other Ca_V_-channels. Likely, Na_V_-like VSDs emerge before sodium selectivity.

## Abstract (web summary)

Low-voltage activated calcium channels (Ca_V_3) regulate cellular excitability by opening in response to modest membrane depolarizations. This study reveals the operation of the voltage-sensing machinery of the human Ca_V_3.1 channel and sheds light on its mechanism of low-voltage sensitivity.

## Introduction

**Fig. 1:**
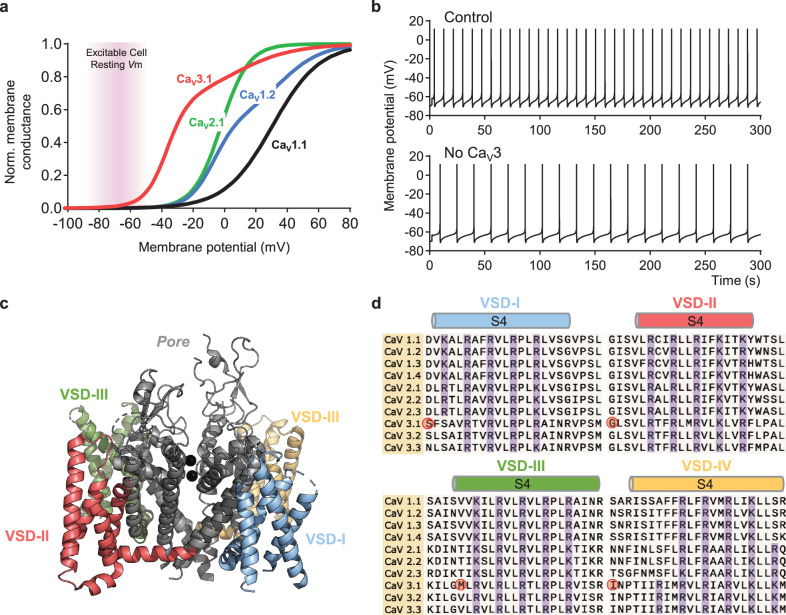
T-type Ca_V_3.1 is a low-voltage activated Ca^2+^ channel. **a** Ca_V_ channels are classified based on their voltage sensitivity, either Low Voltage Activated (LVA, Ca_V_3.x) or High Voltage Activated (HVA, Ca_V_1.x, and Ca_V_2.x). T-type Ca_V_3.1 opens in response to modest depolarizations, allowing Ca^2+^ influx near the resting membrane potential of excitable cells. The reported curves were constructed from the Boltzmann distribution fit parameters in: Ca_V_3.1 (red), Ca_V_2.1 (green)^16^, Ca_V_1.2 (blue)^12^ and Ca_V_1.1 (black)^17^. **b** Action potentials in a modeled entorhinal cortex stellate neuron (ModelDB accession no: 266797)^6^. The simulation is performed in the presence (control, upper trace) and in the absence (No Ca_V_3, bottom trace) of T-type Ca²⁺ conductance. The action potential frequency is 0.12 Hz with T-type Ca²⁺ conductance and decreased to 0.067 Hz in its absence—nearly a 40% reduction. This modeling exercise highlights the critical role of Ca_V_3 channels in the regulation of neuronal firing. **c** Cryo-EM structure of the Ca_V_3.1 (α_1G_) channel (Protein Data Bank ID 6KZO, adapted from ref. ^7^), illustrating the four VSDs that surround and control the central pore. Ca^2+^ ions are shown as black spheres. **d** Sequence alignment of the S4 segments from the ten human Ca_V_ channels. The positively charged residues (Arg, R and Lys, K) within the S4 segment are shaded purple and follow a pattern of a positive amino acid every third residue. These positive residues are highly conserved among all the Ca_V_ channels. Amino acids substituted by Cys used for fluorescence labeling are circled in red. The alignment was obtained using the full-length sequences of all the Ca_V_ channels using the MUSCLE algorithm and selecting the four VSD regions illustrated in the figure.

Low-voltage-activated (LVA) Ca^2+^ channels (Ca_V_3.1, 3.2, 3.3; also known as T-type) are distinct among the voltage-gated calcium channel (Ca_V_) family as they open in response to modest cell membrane depolarizations near the resting membrane potential of excitable cells ( − 70 to − 50 mV)^1,2^. Their peculiar voltage dependence is the hallmark that distinguishes LVA channels from other Ca_V_ channels, which only activate in response to full depolarization and are collectively referred to as high-voltage-activated (HVA) (Fig. 1a). Because of this feature, LVA channels are distinctly suited to support specialized excitability properties, like the pacemaker activity of nodal cardiac myocytes^3^ and the rhythmic firing of thalamic, cerebellar and entorhinal cortex neurons^4–6^ (Fig. 1b).

Despite their recognized physiological relevance, the origin of the low-voltage sensitivity of LVA channels remains a physiological and biophysical mystery. The overall structure of their pore-forming proteins offers no major clue, as both LVA and HVA channels share the typical molecular architecture of voltage-gated channels and, by extension, the same general voltage-sensing mechanism. Accordingly, four voltage-sensing domains (VSDs) surround a central Ca^2+^-selective pore (Fig. 1c)^7–10^; the S4-helix in each VSD incorporates a series of positively charged amino acids (Arg or Lys, Fig. 1d) that are compelled to move in response to changes in membrane potential. These conformational changes within the VSDs are presumably transduced to the pore, driving its opening^11^. The four VSDs in pseudotetrameric Ca_V_ and voltage-gated sodium (Na_V_) channels are not equivalent: each possesses a different number and arrangement of charged amino acids and, indeed, we have previously shown that HVA-channel VSDs are functionally diverse, exhibiting distinct voltage-sensing properties^12–17^. However, the fundamental mechanism of LVA calcium channels voltage dependence remains unknown, and particularly if low-voltage pore opening arises from low-voltage activation of one or more VSDs.

As mentioned above, activation of the VSDs in a channel involves charge movement. This is experimentally detectable as gating currents (*I*_g_)—the composite electrical signal produced by the activation of all VSDs—in channels whose pore ionic conductance has been blocked^18^. Such experiments on LVA channels only deepened the mystery of their voltage dependence, as they showed that most VSD charge moved only by strong depolarization, as in HVA and other voltage-gated channels^19–21^. This presented a conundrum, suggesting that most LVA channels in a membrane can be open while most of their VSDs are yet at rest. This scenario fails to explain LVA channels voltage dependence and appears to violate the causal relationship between VSD activation and pore opening.

More recently, extensive mutagenesis on a cytosolic helix rich in positive charges (S6^Cyto^) did impair voltage-dependent activation in Ca_V_3.3^10^, but the part played by S6^Cyto^ in the voltage-sensing process remains unknown. After all, it is unlikely that a protein segment resolved to be cytosolic directly senses and responds to voltage signals (low or high) across the membrane.

In this study, we use voltage-clamp fluorometry (VCF)^22–25^ on the human Ca_V_3.1 channel (here serving as an archetype of LVA channels) to: (i) unravel the molecular mechanisms underlying their distinct voltage-dependent gating; (ii) understand the paradox of high-voltage VSD activation and low-voltage pore opening; (iii) demystify the role of the S6^Cyto^-helix.

## Results

### All four Ca_V_3.1-channel VSDs activate near the resting membrane potential

**Fig. 2:**
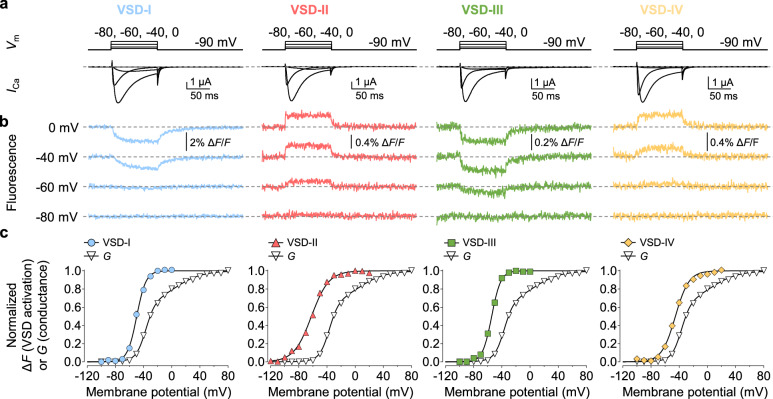
The four Ca_V_3.1 voltage-sensing domains activate at more hyperpolarized membrane potentials compared to pore opening. **a**,** b**, Representative Ca^2+^ currents (*I*_Ca_, **a**) and simultaneously recorded fluorescence signals (**b**) from human Ca_V_3.1 channels reporting local protein structural changes in each VSD. Above the recordings are the voltage-step protocols used. **c** The mean voltage dependence of VSD activation (colored symbols) and WT pore conductance (white triangles). Black lines represent fits to single Boltzmann distributions (VSD) or the sums of two Boltzmann distributions (pore conductance). Note that all VSD activation curves precede pore opening (*G*(V_m_) curve). Data are mean ± SEM. Error bars when not visible are within the symbols. Fitting parameters are reported in Supplementary Table 1. (VSD-I: *n* = 10 cells, VSD-II: *n* = 7 cells, VSD-III: *n* = 9 cells, VSD-IV: *n* = 8 cells, WT *G*(*V*_m_): *n* = 13 cells).

By implementing VCF^22–25^ in human Ca_V_3.1 channels^26^, we simultaneously tracked the voltage-dependent conformational rearrangements of each of the four VSDs and channel function (Ca^2+^ current, *I*_Ca_) under physiologically relevant conditions (2 mM [Ca^2+^]_out_) without blocking the ionic conductance. A cysteine (Cys) was introduced at the extracellular flank of the S4 helix in each VSD of the human Ca_V_3.1 pore-forming protein (α_1G_) to serve as the labeling site for a thiol-reactive fluorophore (Figs. 1d and 2). The channels were expressed in *Xenopus laevis* oocytes and labeled with the environment-sensitive fluorophore MTS-TAMRA, which reports on local conformational changes, *i.e*., VSD activation. The oocytes were then mounted on the cut-open oocyte Vaseline gap voltage-clamp fluorometry (COVG-VCF) system^24,25,27^, to (i) control the membrane potential, (ii) record current, which reflected the state of the channel pore gate; and (iii) record ensemble fluorescence, which reported voltage-evoked conformational changes specific to each labeled VSD.

The exemplary fluorescence recordings demonstrate that the four Ca_V_3.1 VSDs activated with distinct voltage dependence and kinetics (Fig. 2b). The activations of VSD-I and VSD-III resulted in fluorescence decrease (negative fluorescence deflections, or Δ*F*), while the activations of VSD-II and VSD-IV were reported as fluorescence increase (Δ*F* > 0). A straightforward interpretation of this observation is that the active conformations of VSD-I and VSD-III are a more quenching environment for the attached fluorophore relative to the resting conformations; and vice-versa for VSD-II and VSD-IV. Importantly, no voltage-dependent Δ*F* were observed in wild-type channels, WT (with no engineered cysteines) after labeling with MTS-TAMRA (Supplementary Fig. 1a, b), suggesting that endogenous cysteines are not accessible to the fluorophore or do not undergo voltage-dependent movements.

The voltage dependence of pore opening (*G*(*V*_m_)) in wild-type channels had two components. The more negative component had a half-activation potential (*V*_half(1)_) of − 37 ± 0.94 mV and valence (*z*_(1)_) of 4.2 ± 0.27 *e*_0_. The second component had *V*_half(2)_ = 3.4 ± 1.9 mV and *z*_(2)_ = 1.4 ± 0.035 *e*_0_ (Supplementary Fig. 1c and Supplementary Table 1). Overall, the *G*(*V*_m_) of labeled channels carrying Cys mutations for VCF closely recapitulate the functional properties of WT channels (Supplementary Fig. 1c and Supplementary Table 2). Although a small difference in the valence (*z*) of one component of the double Boltzmann fit was observed for Cys mutants in Repeats I and IV (Supplementary Fig. 1c and Supplementary Table 2), the Cys mutants used in this study largely retain WT-like properties.

Remarkably, and in stark contrast to data from *I*_g_ experiments^19–21^, all VSDs activated at more negative voltages relative to pore opening (Fig. 2c). Modest depolarizations ( < −60 mV) engaged the activations of all four VSDs with minimal pore opening (Fig. 2c).

The *V*_half_ of the four VSDs were clustered within a relatively narrow range of ~15 mV, near the resting membrane potential of excitable cells. Specifically, VSD-II activated at the most negative potentials (*V*_half_ = −62 ± 1.2 mV), followed by VSD-III (*V*_half_ = −55 ± 0.84 mV), VSD-I (*V*_half_ = −50 ± 0.95 mV), and VSD-IV (*V*_half_ = −45 ± 0.97 mV) (Fig. 2c and Supplementary Table 1). VSD-I and VSD-III exhibited the steepest voltage dependence (effective valence; *z* ≈ 4 *e*_0_) compared to VSD-II and VSD-IV (*z* ≈ 2 *e*_0_) (Fig. 2c and Supplementary Table 1), indicating that the former are highly sensitive to voltage changes.

**Fig. 3:**
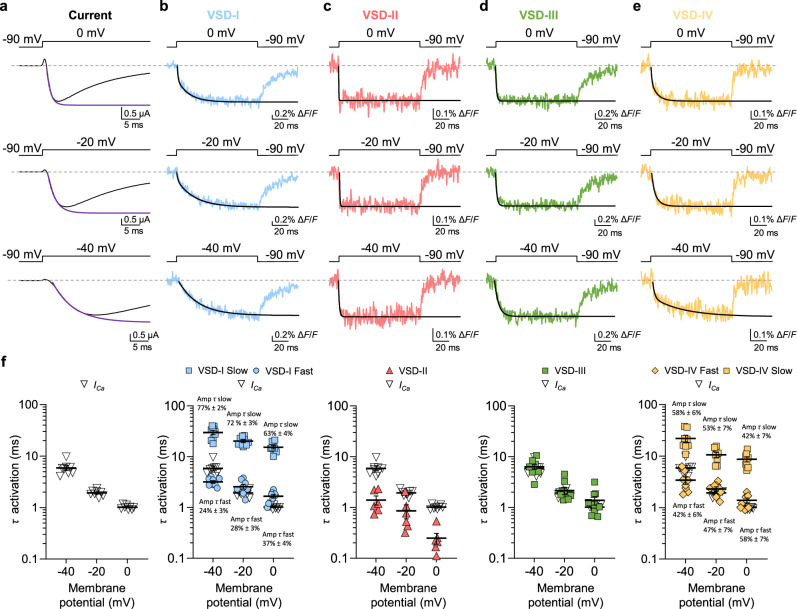
The activation kinetics of all VSDs are compatible with the pore opening. **a** Representative WT Ca_V_3.1 ionic current recordings (black) elicited by depolarizations to − 40 mV, − 20 mV and 0 mV. Superimposed purple lines are the best fits to an exponential function describing ionic current activation. **b–e** Representative fluorescence traces of the four VSDs (in colors). The activation kinetics of each VSD were well described by a single (VSD-II and -III) or double (VSD-I and VSD-IV) exponential function (superimposed black lines). **f** The time constants (*τ*) of ionic current onset (black open triangles) and each VSD activation (colored symbols) at the indicated membrane potentials. For VSD-I and VSD-IV, the relative amplitudes (Amp%) of fast and slow components are reported above and below the respective *τ* values. Note that all VSDs exhibit a time course of activation equal or faster to that of ionic current onset. Data are mean ± SEM. (Current: *n* = 8 cells, VSD-I: *n* = 9 cells, VSD-II: *n* = 6 cells, VSD-III: *n* = 9 cells, VSD-IV: *n* = 8 cells).

Each VSD reacted to depolarizing pulses with distinct kinetics (Fig. 3). VSD-II and VSD-III activated very rapidly with time constants (*τ*) faster or equal to those of the pore opening (at −20 mV, ionic current: *τ* = 1.9  ±  0.091 ms; VSD-II: *τ* =  0.86  ±  0.26 ms; VSD-III: *τ*  =  2.1  ±  0.35 ms; Fig. 3a, c, d, f). In contrast, VSD-I and VSD-IV activations were well fit by the sum of two exponential functions. The fast components of VSD-I (at − 20 mV, *τ*_fast_ = 2.6 ± 0.26 ms, accounting for 28% ± 3% of total amplitude) and VSD-IV (*τ*_fast_ = 2.3 ± 0.23 ms, 47% ± 7% of total amplitude) were also comparable to the rate of channel opening (Fig. 3b, e, f). Both VSDs also displayed a slower fluorescence component. Although we did not further investigate the origin of these signals, they may report slower conformational changes of the protein associated with channel gating other than activation (*e.g*., inactivation).

Altogether, these data supported the premise that the low-voltage dependence of LVA channels emerges from the low-voltage-dependent activation of their four distinct VSDs.

### La^3+^ blocks the Ca_V_3.1 pore and alters the functional properties of select VSDs

Our VCF data partly resolved the longstanding conundrum of LVA-channel molecular physiology, showing that each VSD activation *precedes* pore opening in voltage (Fig. 2). Yet these results were inconsistent with those of *I*_g_ measurements, which showed that activation of the voltage-sensing apparatus *follows* pore opening in voltage^19–21^. To reconcile these diametrically opposite results, we first considered that *I*_g_ measurements require full blockade of the ionic conductance by La^3+^. That is, data about the voltage dependence of pore-opening and VSD-activation are combined from channels under two different conditions: conducting and blocked, respectively. Accordingly, we posited and tested the hypothesis that the blocker La^3+^ alters the voltage-dependent activation of the VSDs, causing a positive shift in the voltage dependence of gating-charge distribution.

**Fig. 4:**
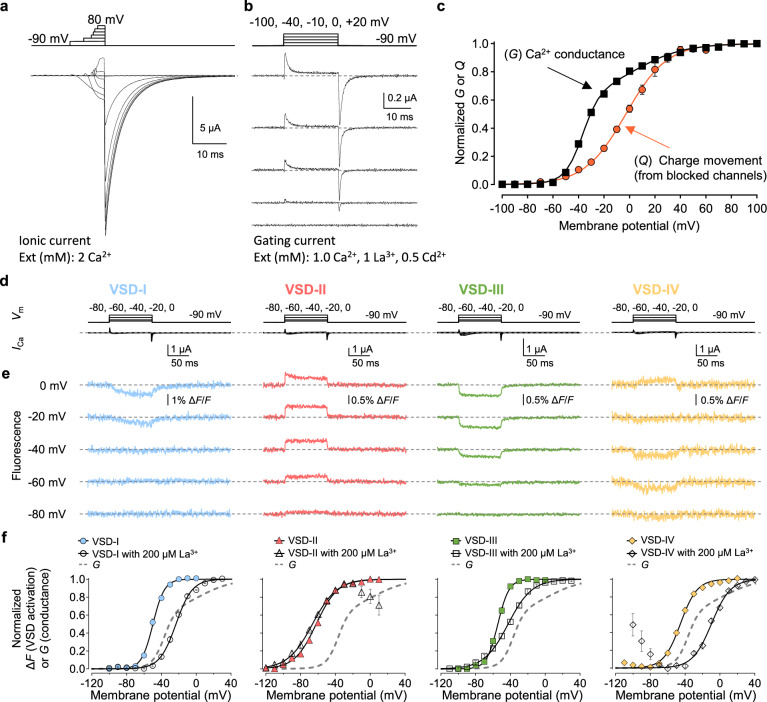
La^3+^ blockade alters Ca_V_3.1 VSD voltage dependence. **a** Representative family of Ca^2+^ currents from oocytes expressing human Ca_V_3.1 channels. The voltage protocol is reported above the current traces. The duration of the depolarizations is progressively shortened not to engage the inactivation process. The tail currents were used to construct the *G*(*V*_m_) curves in (**c**) (black squares, *n* = 13 cells). **b** Ca_V_3.1 gating current recordings from blocked channels (ext. solution with 1 mM La^3+^, 0.5 mM Cd^2+^, *n* = 4 cells). **c** Voltage dependence of channel opening (*G*, black squares) and charge movement (*Q*, orange circles): note that under these experimental conditions the *Q*(*V*_m_) curve (all-VSD combined activation) is to the right of the *G*(*V*_m_) curve (pore open probability). These findings were first reported by ref. ^19^. Fitting parameters are reported in Supplementary Table 1. **d**,** e** Representative Ca^2+^ currents (*I*_Ca_, **d**) and simultaneously recorded fluorescence (**e**) signals (reporting local protein structural changes in each VSD) from Ca_V_3.1 channels in the presence of 200 µM La^3+^. Above the recordings are the step voltage protocols used. **f** The mean voltage dependence of VSD activations without (from Fig. 2, filled symbols) or with 200 µM La^3+^ (black open symbols). Continuous black lines represent fits to single Boltzmann distributions. Dashed gray lines are wild-type *G*(*V*_m_) curve fits (from **c**). Note that in La^3+^-blocked Ca_V_3.1 channels, VSD-I, -III and -IV activate at more depolarized membrane potentials compared to conducting channels. Data are mean ± SEM, error bars when not visible are within the symbols. Fitting parameters and *p-*values are reported in Supplementary Table 3, two-tailed unpaired Student’s *t* tests. (VSD-I unblocked: *n* = 10 cells, with La^3+^: *n* = 4 cells; VSD-II unblocked: *n* = 7 cells, with La^3+^: *n* = 5 cells; VSD-III unblocked: *n* = 9 cells, with La^3+^: *n* = 7 cells; VSD-IV unblocked: *n* = 8 cells, with La^3+^: *n* = 5 cells; WT unblocked *G*(*V*_m_): *n* = 13 cells).

First, we reproduced the depolarized charge movement of Ca_V_3.1-channels by measuring *I*_g_ during blockade by La^3+^ and Cd^2+^ (Fig. 4b-c). In line with previous observations^19–21^, the charge displaced by the activation of all VSDs occurred at voltages more positive than channel opening (*Q*(*V*_m_) *V*_half_ = − 2.2 ± 2.1 mV and *G*(*V*_m_) = −31 ± 1.0 mV, respectively).

Using VCF, we found that just 200 µM La^3+^ differentially affected the voltage dependence of Ca_V_3.1-channel VSDs (Fig. 4d–f). VSD-I and VSD-IV were the most affected by the blocker: their activation curves shifted in the positive direction by 26 ± 1.5 mV and 35 ± 1.6 mV, respectively (Fig. 4f and Supplementary Table 3). Of note, the voltage-dependent activations of VSD-I and VSD-IV in channels blocked by La^3+^ occurred at more positive voltages than pore opening, measured previously in the absence of La^3+^. VSD-III activation was also significantly perturbed, with a positive shift of 12 ± 1.6 mV and a reduction in voltage sensitivity by over 50% (control: *z* = 4.1 ± 0.11 *e*_0_, La^3+^: *z* = 1.8 ± 0.12 *e*_0_) (Fig. 4f and Supplementary Table 3).

VSD-II voltage-dependent activation was largely unaffected (Fig. 4d–f and Supplementary Table 3). Of note, the activations of VSD-II and VSD-IV also exhibited additional fluorescence components, at positive and negative potentials, respectively, so their voltage dependence curves were no longer monotonic (Fig. 4f). This suggested a modification of these Ca_V_3.1 VSDs by La^3+^, perturbing their active and resting states, respectively.

Taken together, these data supported our hypothesis and provided evidence that the depolarized charge movement reported by *I*_g_ measurements in blocked channels is a misleading experimental result, caused by the perturbation of selected VSDs by La^3+^ blockade.

### The positive charges of the S6^Cyto^*-*helix support the roles of VSD-I, VSD-III and VSD-IV in low-voltage activation

**Fig. 5:**
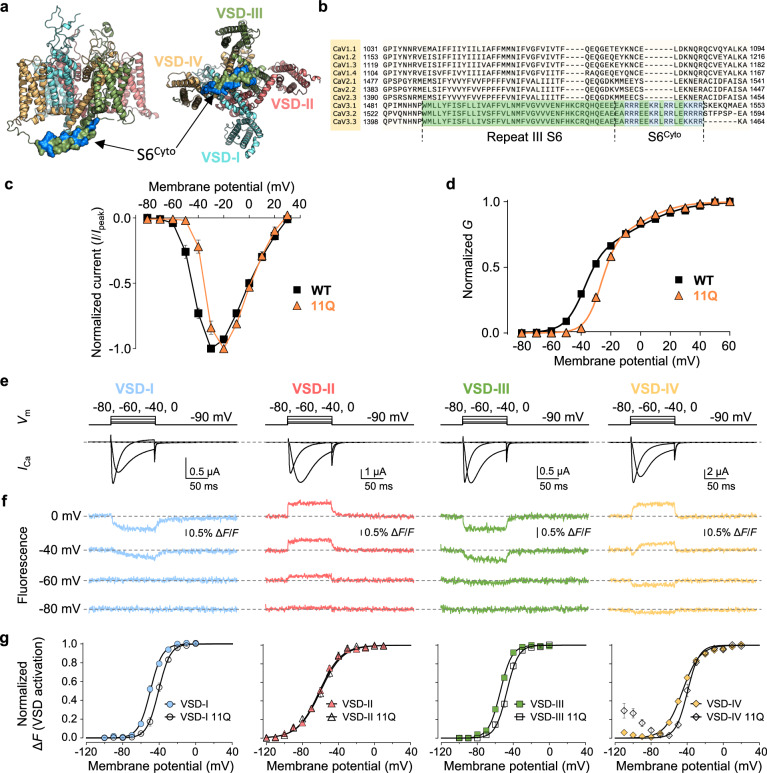
Neutralization of positive charges in S6^Cyto^ perturbs Ca_V_3.1 channel opening and VSD activation. **a** CryoEM structure of Ca_V_3.3 (side and bottom views, Protein Data Bank ID 7WLI,^10^). Ca_V_3.3 contains a long, bent S6 helix in Repeat-III protruding into the cytosol, named S6^Cyto^ (shown as a surface). S6^Cyto^ is characterized by a series of positively charged residues (blue). **b** Sequence alignment of Repeat III S6 helix of all human Ca_V_ channels (positively charged residues in blue). Note that S6^Cyto^ is exclusively and highly conserved among LVA channels. **c** Normalized peak current *vs* voltage relationship in WT channels, and channels where the eleven S6^Cyto^ positive residues were substituted by neutral glutamines (11Q). Data are mean ± SEM. (WT: *n* = 12 cells, 11Q: *n* = 6 cells). **d** Voltage dependence of Ca_V_3.1 activation in WT or 11Q. Neutralization of positively charged amino acids in the latter impaired channel opening, causing a depolarizing shift of the conductance voltage dependence. Data are mean ± SEM. (WT *G*(*V*_m_): *n* = 13 cells, 11Q *G*(*V*_m_): *n* = 6 cells). **e**,** f** Exemplary current (**e**) and fluorescence (**f**) recordings in 11Q mutant. **g** The mean voltage dependence of VSD activations in 11Q mutant (black open symbols) and WT channels (from Fig. 2, filled symbols). Black lines represent fits to single Boltzmann distributions. Note that the 11Q mutation perturbed VSD-IV activation as revealed by a new component in the fluorescence voltage dependence at negative membrane potentials (< − 90 mV). The activations of VSD-I and VSD-III were also modified, as revealed by a more depolarized voltage dependence of VSD activation compared to the WT. Data are mean ± SEM, two-tailed unpaired Student’s *t* tests. When not visible, the error bars are within the symbols. Fitting parameters and *p-*values are reported in Supplementary Table 4. **(**VSD-I WT: *n* = 10 cells, 11Q: *n* = 6 cells; VSD-II WT: *n* = 7 cells, 11Q: *n* = 4 cells; VSD-III WT: *n* = 9 cells, 11Q: *n* = 7 cells; VSD-IV WT: *n* = 8 cells, 11Q: *n* = 5 cells).

Several intracellular regions of the Ca_V_3.1 have been shown to contribute to the channel gating^28–30^. Interestingly, a recent study on Ca_V_3.3 postulated that the long S6 helix of Repeat-III protruding into the cytosol (S6^Cyto^) is critical for LVA-channel low-voltage opening^10^ (Fig. 5a). S6^Cyto^ incorporates a series of positively charged amino-acids (^1530^RRREEKRLRRLEKKRR^1545^), it is highly conserved among LVA channels and absent in the HVA Ca_V_ channels (Fig. 5b)^10^. To understand the mechanism by which this region supports low-voltage sensitivity, we substituted all 11 positively charged amino acids (R and K) with uncharged glutamines (Q), neutralizing the charge of the Ca_V_3.1 S6^Cyto^: we called this mutant 11Q.

We found that this charge neutralization (11Q) impaired Ca_V_3.1 activation, revealed by a positive shift of the peak of the current-voltage relationship (Fig. 5c) and about +10 mV shift in the voltage dependence of channel activation (Fig. 5c, d and Supplementary Table 4). Similar results have been observed in another T-type channel (Ca_V_3.3)^10^, suggesting a similar mechanism across LVA channels. As in the case of La^3+^-blocked channels (Fig. 4), the voltage-dependent properties of VSD-II were not affected by the 11Q mutations (Fig. 5e–g and Supplementary Table 4). This suggests that VSD-II activation is not (or is weakly) involved in channel opening, as discussed below. Nevertheless, the impairment of pore opening was positively associated with a significant perturbation of the voltage-dependent activation of three VSDs (Fig. 5e–g).

Specifically, the activations of VSD-I and VSD-III were inhibited, both requiring stronger depolarization by ~ 10 mV relative to wild-type channels (Supplementary Table 4). Yet the VSD most affected by S6^Cyto^ charge neutralization was VSD-IV. In wild-type channels, VSD-IV activation was reported as positive Δ*F* (Fig. 2b) with a single, monotonic, voltage-dependent distribution (Fig. 2c). In Ca_V_3.1-11Q, VSD-IV exhibited an additional voltage-dependent fluorescence component of opposite sign (Δ*F* < 0) (Fig. 5f, g). While this type of composite fluorescence signal precludes the parameterization of VSD-IV voltage dependence, it is consistent with a structural perturbation, more profound than a mere inhibition of the activation transition. Specifically, we propose that S6^Cyto^ neutralization allowed VSD-IV to adopt a distinct deep resting conformation, discussed below.

## Discussion

### LVA-channel VSDs are tuned to respond within a narrow range of modest depolarizations

Due to their low-voltage sensitivity, LVA Ca_V_ channels control voltage-evoked Ca^2+^ influx near the cell resting membrane potential, at voltages where HVA channels have near-zero probability to open. Thus, the primary role of LVA channels is to tune cell excitability *via* the depolarizing effect of Ca^2+^ influx rather than contribute to intracellular Ca^2+^ dynamics or signaling, which seems to be a prerogative of HVA channels. To shed light on the mechanisms of the exquisite sensitivity of LVA channels to modest membrane depolarizations, we investigated the activity of the four individual VSDs of Ca_V_3.1 channels. We discovered two seminal features that set LVA VSDs apart from those of the HVA Ca_V_ channels investigated so far (*i.e*., Ca_V_1.1^14,17^, Ca_V_1.2^12,13^, Ca_V_2.1^16^ and Ca_V_2.2^15^).

First, the four VSDs of Ca_V_3.1 activate in response to small depolarizations, from − 60 to − 45 mV. While HVA channels may also possess some VSDs that activated by modest depolarizations (or even have a high probability of being active at resting membrane potentials), these are not thought to make a significant contribution to pore opening. Instead, HVA channels also possess VSDs that activate by stronger depolarizations, and it is they that drive high-voltage pore opening^12,14–17^.

**Fig. 6:**
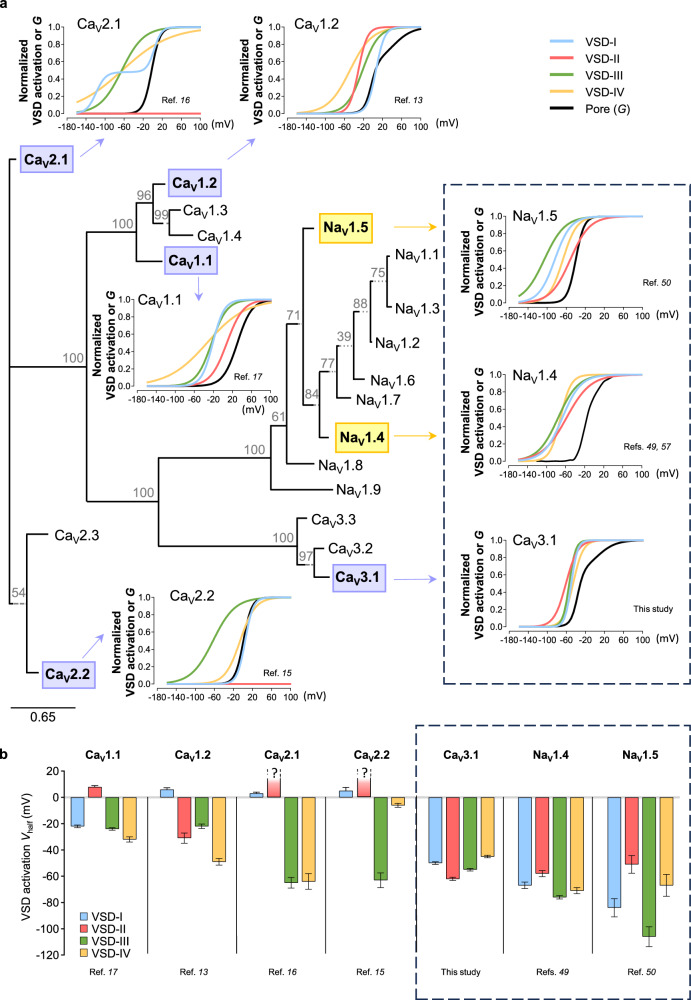
The four VSDs of the LVA Ca_V_3.1-channel are evolutionarily closer to Na_V_ channels. **a** Phylogenetic tree of Ca_V_ and Na_V_ channels was constructed from the sequence alignment of the VSD S1-S4 regions using the maximum likelihood approach (see Supplementary Fig. 3a for amino-acids used for the phylogenetic tree). Node support values resulting from 1000 bootstrap replicates are indicated in gray, and the scale bar indicates the number of amino acid substitutions per site. Insets show the voltage dependence of VSD activation (VSD-I: blue; VSD-II: red; VSD-III: green; VSD-IV: orange) and pore opening (black) in Ca_V_1.1^17^, Ca_V_1.2^13^, Ca_V_2.1^16^, Ca_V_2.2^15^, Ca_V_3.1 (this study), Na_V_ (Na_V_1.4^49^, pore^57^ and Na_V_1.5^50^) channels. Ca_V_3.1 channels share a common ancestor with Na_V_ channels and are more closely related to Na_V_ than to other Ca_V_1 and Ca_V_2 channels. Note that the four VSDs of Ca_V_3.1, Na_V_1.4 and Na_V_1.5 channels display high similarity in their voltage-dependent properties (boxed area) as further highlighted in (**b**). **b** The plot shows the half-activation potential of the individual VSDs in Ca_V_ and Na_V_ channels from all currently available VCF studies (Ca_V_1.1^17^, Ca_V_1.2^13^, Ca_V_2.1^16^, Ca_V_2.2^15^, Ca_V_3.1 (this work), Na_V_ (Na_V_1.4^49^ and Na_V_1.5^50^). Note that in Ca_V_3.1 and Na_V_ channels, all four VSDs activate at hyperpolarized membrane potentials near the resting membrane potential of excitable cells (boxed area). On the other hand, the HVA channels possess VSDs with a more heterogeneous voltage dependence with some of the sensors displaying a positive activation *V*_half_ or even failed to respond to depolarization up to + 200 mV (denoted with?). Data are mean ± SEM.

Second, the Ca_V_3.1 VSDs are functionally homogeneous: they all operate within a narrow range of membrane potentials spanning a mere 15 mV (Fig. 2c, Supplementary Table 1 and Fig. 6). By contrast, HVA-channel VSDs are more heterogeneous, spanning 50 mV in physiologically relevant channel complexes (Fig. 6), and even more so in the absence of necessary regulatory subunits^13^. This feature aligns with their physiological role, as LVA channels are expected to activate within a few millivolts of the resting membrane potential, shaping the excitation properties of neurons and pacemaker myocytes.

### La^3+^ blockade alters VSD voltage dependence

It was previously observed that La^3+^, besides blocking the pore, also reduced the size of Ca_V_3.1 gating currents^31^. Here, we present compelling evidence that the voltage dependence of specific VSDs is profoundly altered in La^3+^-blocked Ca_V_3.1 channels (Fig. 4). La^3+^ binds in the selectivity filter and blocks Ca^2+^ influx^32,33^. The altered VSD activity can be due to an allosteric effect from the pore, surface charge screening or a direct interaction of La^3+^ with the VSDs^34^. Regardless of the exact mechanism of this perturbation (irrelevant to the conclusions of this study), VSD modification is an undesirable effect of the blocker when used to measure LVA channels gating currents to evaluate VSD operation. The result demonstrates that the apparent depolarized VSD voltage dependence measured by gating currents is most likely caused by direct or indirect interactions of the obligatory blocker with the VSDs and refutes the assumption that pore blockade leaves the VSDs function untouched. VCF modifications can also affect VSD activity and pore opening: however, its ability to monitor both at the same time provides the opportunity to refine the VCF strategy (by trialing alternative labeling positions and fluorophores) and chose that with the least overall perturbation.

### A structural model for the S6^Cyto^ contribution to low-voltage sensitivity

The cytoplasmic extension of the S6-helix of Repeat-III (S6^Cyto^) is a distinct feature of LVA channels^10^. Examining its contribution to low-voltage activation, we found that it selectively remodels the voltage dependence of three VSDs, partially supporting low-voltage sensitivity (Fig. 5). Interestingly, the S6^Cyto^ was resolved to be most proximal to VSD-IV, which we found to be the most perturbed. Combining this structural information with our VCF data, we consider the following model.

First, with an intact S6^Cyto^, VSD-IV voltage-dependent activation occurs with a single distribution (Fig. 2c and Supplementary Fig. 2). The most straightforward interpretation of this is that it can adopt two conformational states: resting at negative voltages and active at slightly more depolarized voltages. The fluorophore labeling VSD-IV was more quenched at the resting state compared to the active state, so depolarization produced positive Δ*F*. The activity curve saturated by − 80 mV (Fig. 2c, VSD-IV and Supplementary Fig. 2a, b), suggesting that VSD-IV is in the resting conformation at more negative potentials.

Second, neutralization of the 11 positive charges in S6^Cyto^ resulted in an additional fluorescence component of the signal from VSD-IV: hyperpolarizations from − 90 mV, produced positive Δ*F* (Fig. 5f, g), suggesting that, in the absence of an intact S6^Cyto^, VSD-IV adopts a distinct, third conformation, whereby the fluorophore is less quenched than in the resting state; this state occurs at more negative potentials (Fig. 5g); *i.e*., it is a deep resting conformation (Supplementary Fig. 2a and c).

A reasonable interpretation of this effect is that the positively charged S6^Cyto^ electrostatically opposes occupancy of the VSD-IV deep resting state, keeping VSD-IV only one step away from the fully activated state, facilitating channel opening. A structural interpretation of the VCF data is provided in Supplementary Fig. 2.

### VSD-II appears functionally uncoupled from the Ca_V_3.1 pore

The effects of S6^Cyto^ neutralization on distal VSD-I and VSD-III can be rationalized as allosteric, *via* the functional interactions between their activated conformations and the open pore. On the other hand, VSD-II remains unmodified after S6^Cyto^ neutralization: the resilience of VSD-II activation to a significant gating perturbation (11Q) implies its dissociation from the process of voltage-dependent opening. These results lend support to previous studies that concluded that the Ca_V_3.1 Repeat-II is unlikely to contribute to pore opening^35^, and stressed the preeminent role of VSD-I and -IV to voltage-dependent gating of Ca_V_3.1^36,37^. Interestingly, while VSD-II appears largely dispensable for gating in Ca_V_3.1, it assumes a more prominent role in the opening of the LVA Ca_V_3.3 subtype, as reported by McArthur and colleagues^38^.

Thus, VSD-I, VSD-III and VSD-IV, which appear to drive pore opening, should be considered ideal targets for drug-development efforts against Ca_V_3.1-associated channelopathies like cerebellar ataxia^39–43^, or to correct dysregulated excitability of the cerebello-thalamo-cortical circuit, as in essential tremor and seizures^44–46^.

### Inferring Ca_V_/Na_V_ evolution from VSD functional studies and sequence analysis

In 1984, B. Hille suggested that with the emergence of the early animal nervous system, voltage-gated sodium channels (Na_V_) evolved from Ca_V_ channels^47^, and a recent hypothetical phylogenetic tree has suggested that Na_V_ and LVA Ca_V_-channels diverged from a common ancestor^48^.

The properties of VSDs provide a further means to explore the evolution of these channels.

The VSDs of Ca_V_3.1 have a noteworthy similarity with the VSDs of Na_V_ channels studied by VCF to date (Na_V_1.4^49^ and Na_V_1.5^50^): they all display hyperpolarized activation *V*_half_ (Fig. 6). By contrast, the VSD properties of HVA Ca_V_ channels resolved so far (Ca_V_1.1^14,17^, Ca_V_1.2^12,13^, Ca_V_2.1^16^ and Ca_V_2.2^15^) activate at a broader range of *V*_half_ (Fig. 6b). While this observation is yet tentative, pending the characterization of all Ca_V_ and Na_V_ VSDs, it prompted us to examine the evolutionary hypothesis of Ca_V_ and Na_V_ channels, in light of the current theory of Na_V_ evolving from Ca_V_^47,51–53^. We assembled the amino-acid sequences of all nineteen Ca_V_ and Na_V_ channels and subjected them to a preliminary phylogenetic analysis using a maximum-likelihood approach. Whether we used the full-length sequence (Supplementary Fig. 3b), the VSDs (Fig. 6a), or just the S4 regions (Supplementary Fig. 3c), a similar tree was produced: specifically, (i) Na_V_ and LVA Ca_V_ channels shared a common ancestor as supported by the bootstrap values; and (ii) LVA are more closely related to Na_V_ than to all other Ca_V_ channels (Fig. 6 and Supplementary Fig. 3).

Furthermore, the functional similarity with regards to LVA Ca_V_ and Na_V_ in controlling or regulating neuronal excitability presents corroborating evidence for evolutionary relatedness. The overlap of some aspects of VSD functional properties and sequence analyses (Fig. 6) agree with the theory that LVA Ca_V_ and Na_V_ share a common ancestor, as proposed 20 years ago^47^.

Such analysis has the implication that low-voltage-activating VSDs emerged in LVA Ca_V_-channels prior to the Ca^2+^ to Na^+^ switch in ionic selectivity. The evolutionary advance of this change, which allowed separation of electrical excitation and Ca^2+^ signaling, is an intriguing area of future study.

In summary, this study offered a surprising view of the operation of the voltage-sensing machinery of a functional, human LVA Ca_V_ channel and sheds light on the mechanistic origin of its low-voltage activation. Under physiologically relevant conditions, all four VSDs of Ca_V_3.1 operate at hyperpolarized membrane potentials and activate at voltages more negative than pore opening. The blocker La^3+^ impairs the activation of the four VSDs in Ca_V_3 channels, accounting for the counter-intuitive results of the *I*_g_ method, thereby conflating results from blocked and unblocked channels. The distinct LVA-channel structural motif S6^Cyto^ supports the low-voltage activation of the four VSDs. The experiments are also consistent in dissociating VSD-II activation from the process of voltage-dependent channel opening. In addition to elucidating the molecular physiology of voltage sensing, the VSD functional properties also inform on channel evolution, supporting the close relationship of LVA Ca_V_ and Na_V_ channels.

## Methods

### Ethical statement

The animal protocol was approved by the UCLA Institutional Animal Care and Use Committee and conformed to the Guide for the Care and Use of Laboratory Animals published by the U.S. National Institutes of Health.

### Neuron action potential simulation

We used the entorhinal cortex layer II stellate neuron model developed by Topczewska et al.^6^ (ModelDB accession no: 266797), implemented in the NEURON simulation environment. The action potential simulations were obtained from the soma using a 110-pA current injection.

### Molecular biology

The human *CACNA1G* cDNA (α_1G_, UniProt accession no. O43497-2) was subcloned from hα1Ga-pDsRed (Addgene #45811; http://n2t.net/addgene:45811; RRID:Addgene_45811; a gift from Edward Perez-Reyes^26^) to the pMAX vector for in vitro transcription and oocyte expression. For voltage clamp fluorometry, clones with cysteines substituted at the extracellular flank of the S4 helix of each VSD (S175C, G829C, M1353C or I1669C for VSDs I-IV, respectively) were generated by GenScript Biotech (Piscataway, NJ, USA) and confirmed by sequencing. The plasmid was linearized using PacI (New England Biolabs, Ipswich, MA, USA) and transcribed to cRNA in vitro (AmpliCap-Max™ T7 High Yield Message Maker Kit, CellScript).

### *Xenopus* oocytes isolation

Oocyte lobes were surgically harvested from *Xenopus laevis* and enzymatically defolliculated, as previously described^14^. Mature (stage V-VI) oocytes were injected with 50 nl cRNA (1 µg/µl) and incubated for 4–6 days at 18 °C in either SOS (in mM: 100 NaCl, 2 KCl, 1.8 CaCl_2_, 1 MgC1_2_, and 5 HEPES, pH 7, with 100 µ/ml penicillin, 100 µg/ml streptomycin, and 50 µg/ml gentamicin) or in a solution composed of 50 % Leibovitz’s L-15 (Corning cellgro), 47.5 % H_2_O, 10 % heat-inactivated horse serum (HyClone), 100 µ/ml penicillin, 100 µg/ml streptomycin, and 100 µg/ml amikacin (Cayman Chemical Company).

### Cut-open oocyte vaseline gap - voltage-clamp fluorometry (COVG-VCF)

Voltage-clamp fluorometry (VCF)^22^ was performed at room temperature using the Cut-open oocyte Vaseline gap (COVG), implemented for epifluorescence measurements^23–25,27^.

Oocytes were incubated with thiol-reactive fluorophore sensitive to environmental changes (20 μM MTS-TAMRA, Santa Cruz) in a depolarizing solution (in mM: 120 K-Methanesulfonate (MES), 2 Ca(MES)_2_, and 10 HEPES, pH = 7.0) for 5 minutes on ice. Fluorescence changes and ionic currents were acquired simultaneously from the same membrane area. External solution (mM) contained 120 NaMES, 2 Ca(MES)_2_, 10 HEPES (pH = 7.0). Internal solution (mM) contained 120 K-Glutamate, 10 HEPES (pH = 7.0). Intracellular micropipette solution (mM) was composed of 2700 NaMES, 10 NaCl and 10 HEPES (pH = 7.0).

Just before mounting, each oocyte was injected with 100 nl of 80 mM BAPTA•4 K, 10 mM HEPES (Invitrogen) (pH = 7.0) to prevent activation of endogenous Ca^2+^- and Ba^2+^-dependent Cl^−^ channels^54^. To investigate the effect of La^3+^, the VCF extracellular solution was supplemented with 0.2 mM LaCl_3_. Experiments were performed at room temperature. Ionic current and fluorescence were filtered at 1/5 of the sampling frequency (typically 2 kHz).

### Cut-open oocyte vaseline gap – gating currents (COVG-*I*_g_)

Experiments were performed in oocytes expressing wild-type Ca_V_3.1 pre-injected with BAPTA, as above. To isolate gating currents, the extracellular solution containing (mM) 120 NaMES, 1 Ca(MES)_2_, 10 HEPES (pH = 7.0) was supplemented with 1 LaCl_3_, 0.5 CdCl_2_, and 0.1 ouabain to block Ca_V_3.1 ionic currents and the Na^+^/K^+^ pump. The same intracellular and electrode solutions were used as above. Experiments were performed at room temperature. Gating currents were filtered at 1/5 of the sampling frequency.

### Data analysis

The voltage dependence of channel opening (proportional to macroscopic conductance, *G*(*V*)) was obtained from the peak tail current at − 90 mV and plotted against the test potential. Its voltage dependence was empirically characterized by fitting to the sum of two Boltzmann functions as:

1 $$G({V}_{{{{\rm{m}}}}})={\sum}_{i=1}^{2}\frac{{w}_{i}}{1+\exp [\frac{{z}_{i}F}{{RT}}({V}_{{{{\rm{half}}}},i}-{V}_{{{\rm{m}}}})]}$$

where *w* was the relative weight such that *w*_1_ + *w*_2_ = 1, *z* was the valence, F and R were the Faraday and gas constants, respectively, *T* was the absolute temperature, *V*_half_ was the half-activation potential, and *V*_m_ was the test membrane potential. The fitting parameters are reported in Supplementary Table 2.

Fluorescence *vs* voltage curves (Δ*F*(*V*)) were constructed by plotting the amplitude of the fluorescence deflection (Δ*F*) at the end of 100-ms test pulses against the test membrane potential. Δ*F* from VSD-I and VSD-III were inverted before analysis. Data points were fit to a Boltzmann equation in this form:

2 $$\Delta F\left({V}_{{{{\rm{m}}}}}\right)=\,\frac{\Delta {F}_{\max }-\Delta {F}_{\min }}{1+\exp \left[\frac{z{{{\rm{F}}}}}{{{{\rm{R}}}}T}\left({V}_{{{{\rm{half}}}}}-{V}_{{{{\rm{m}}}}}\right)\right]}+\Delta {F}_{\min }$$

where Δ*F*_max_ was the maximum Δ*F*, and Δ*F*_min_ was the minimum Δ*F* and *z* was the effective valence. The fitting parameters are reported in Supplementary Tables 1, 3, & 4.

Fluorescence and current kinetics were fitted to a single (*j* = 1), or the sum of two (*j* = 2), exponential function(s):

3 $$f\left(t\right)=B+{\sum}_{i=1}^{j}{A}_{i}\exp \left(-t/{\tau }_{i}\right)$$

where *B* was the baseline, *A* was the amplitude, *t* was time, and *τ* was the time constant.

Charges *vs* voltage curves (*Q*(*V*)) were constructed by plotting the time integral of the on-gating currents against the test membrane potential. Data points were fit to a Boltzmann equation in this form:

4 $$Q\left({V}_{{{{\rm{m}}}}}\right)=\,\frac{{Q}_{\max }-{Q}_{\min }}{1+\exp \left[\frac{z{{{\rm{F}}}}}{{{{\rm{R}}}}T}\left({V}_{{{{\rm{half}}}}}-{V}_{{{{\rm{m}}}}}\right)\right]}+{Q}_{\min }$$

where *Q*_max_ was the maximum charge and *Q*_min_ was the minimum charge. The fitting parameters are reported in Supplementary Table 1.

All curve fitting was performed by least squares using Solver in Microsoft Excel.

### Phylogenetic Tree

Reference human Ca_V_ and Na_V_ channel protein sequences (UniProt accession no.: *CACNA1S*: Q13698*, CACNA1C*: Q13936-1, *CACNA1D*: Q01668-1, *CACNA1F*: O60840-1, *CACNA1A*: O00555-8, *CACNA1B*: Q00975-1, *CACNA1E*: Q15878-1, *CACNA1G*: O43497-2, *CACNA1H*: O95180-1, *CACNA1I*: Q9P0X4-1, *SCN1A*: P35498-1, *SCN2A*: Q99250-1, *SCN3A*: Q9NY46-1, *SCN4A*: P35499, *SCN5A*: Q14524-1, *SCN8A*: Q9UQD0-1, *SCN9A*: Q15858-1, *SCN10A*: Q9Y5Y9, *SCN11A*: Q9UI33-1) nineteen in all, were utilized for alignment with the program SeaView 5.0.5^55^. Accession numbers and amino acids used for the alignment are listed in Supplementary Fig. 3a. Three separate sequence profiles were used: the full-length sequence; the individual VSD (S1-S4), and S4 sequences. Each separate alignment was utilized for maximum likelihood phylogenetic tree generation using the online program IQ-TREE (http://iqtree.cibiv.univie.ac.at). Output for each tree in Newick format was then tree assembled with the online program ETEToolkit^56^ (http://etetoolkit.org/treeview/). All trees generated were qualitatively the same, node support was assessed *via* bootstrapping with 1000 replicates. The VSD (S1–S4) tree is shown in Fig. 6, and comparative trees of full-length sequence and the VSD S4 are shown in Supplementary Fig. 3b, c.

### Statistical analysis

Data are represented as mean ± SEM, and *n* is the number of cells tested. *p-*values were calculated using two-tailed unpaired Student’s *t* tests or one-way analysis of variance (ANOVA) followed by Dunnett’s multiple comparisons.

Statistical analyses were performed with Microsoft Excel and GraphPad Prism.

### Reporting summary

Further information on research design is available in the Nature Portfolio Reporting Summary linked to this article.

## Supplementary information

Supplementary Information

Reporting Summary

Transparent Peer Review file

## Source data

Source Data

## Data Availability

All data are included in the main manuscript and related supplementary files. Previously published structures and related PDB codes used in the manuscript are: 6KZO and 7WLI. Source data are provided in this paper.
