# Supplementary material for: The four voltage-sensing domains of T-type calcium channels activate near the resting membrane potential
Source: Nat Commun. 2026 May 16;17:6506. doi: 10.1038/s41467-026-73077-1 (PMC13377093; doi:10.1038/s41467-026-73077-1)
Supplement: Supplementary file 1 — Supplementary Information [file 41467_2026_73077_MOESM1_ESM.pdf]

The Four Voltage-Sensing Domains of T-Type Calcium Channels Activate Near the Resting Membrane Potential

Marina Angelini, Moira McVicar, Savana Maxfield, Milosz Sokolowski, Sanjana Narang, Kyle Scranton, S. Suheda Yasarbas, Nicoletta Savalli, Scott A. John, Andreas Schwingshackl, Alan Neely, Antonios Pantazis, Michela Ottolia, Riccardo Olcese

SUPPLEMENTARY FIGURES & TABLES

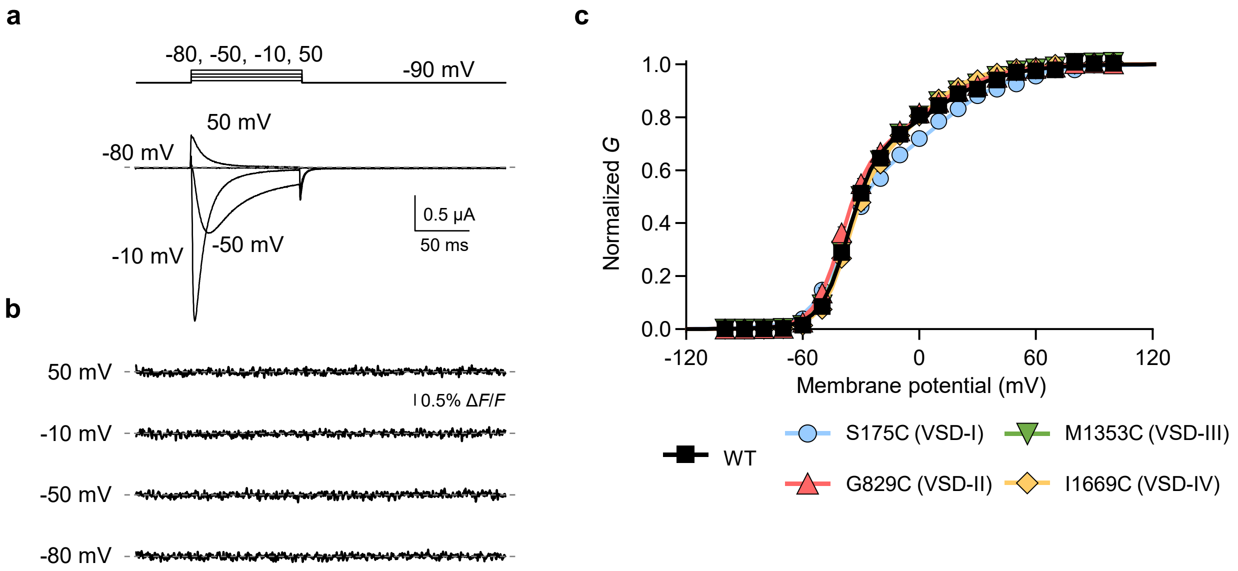

Supplementary Fig. 1: WT Cav3.1 does not elicit voltage-dependent fluorescence changes when labeled with a thiol-reactive MTS-TAMRA fluorophore, and MTS-TAMRA-labeled Cys mutants retain the same voltage dependence as Cav3.1 WT.

a, Representative Ca^{2+} current recordings from an oocyte expressing WT Cav3.1 channel (without engineered cysteines) incubated with MTS-TAMRA. The simultaneously recorded fluorescence is shown in **b**. Note the absence of voltage-dependent changes in the fluorescence recordings (n = 5 cells).

c, Mean voltage dependence of channel opening ($G(V_m)$) for WT (n = 13 cells) and MTS-TAMRA-labeled Cys mutants (S175C n = 10 cells, G829C n = 7 cells, M1353C n = 9 cells, I1669C n = 8 cells). Note that the Cys mutations did not substantially alter the voltage dependence of pore opening compared to WT channel activation. Lines in **c** are fits to double Boltzmann distributions. Fitting parameters are reported in Supplementary Table 2. Data are represented as mean ± SEM, error bars when not visible are within the symbols.

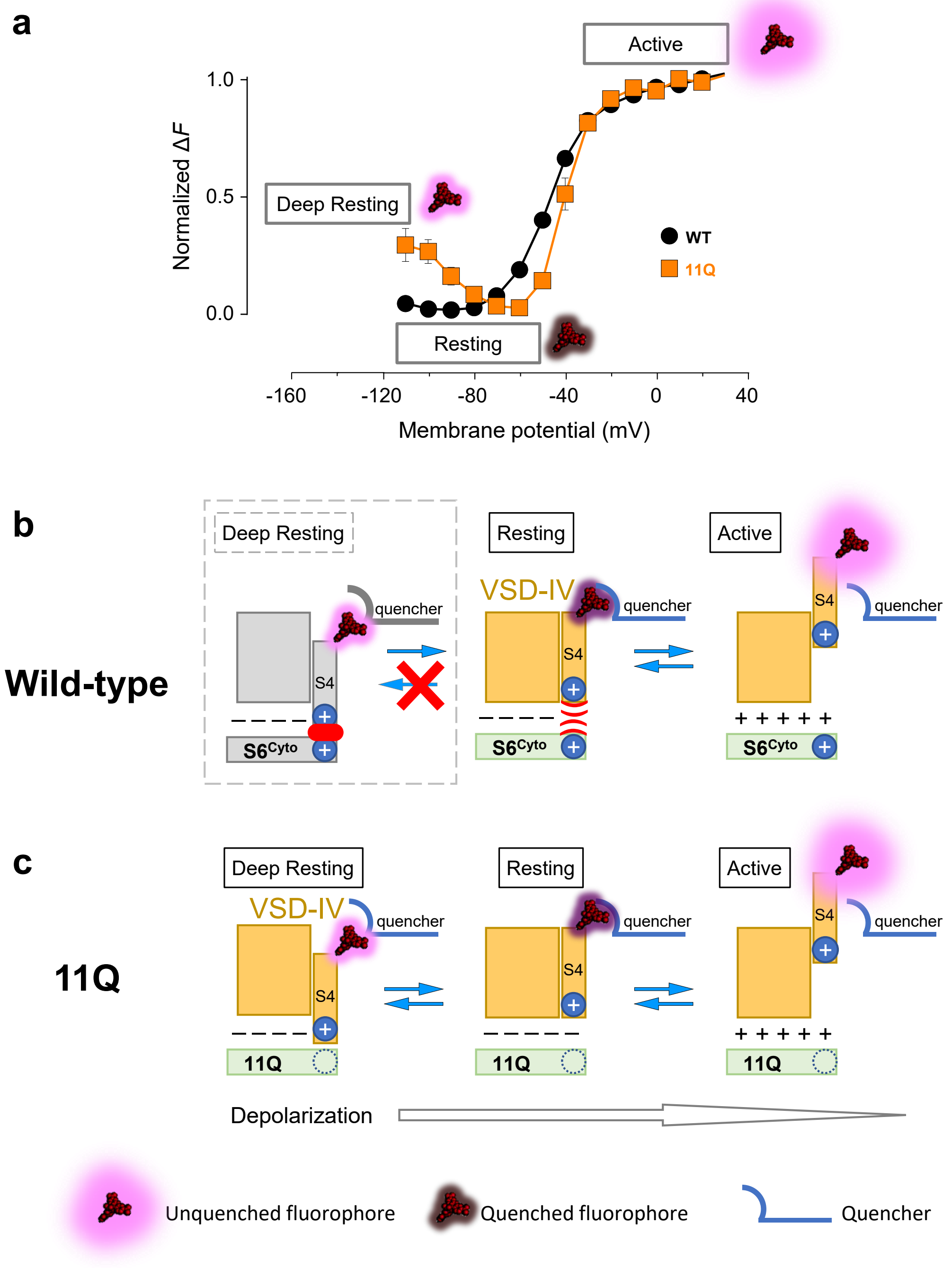

Supplementary Fig. 2: Positive charges in S6^{Cyto} prevent a deep resting state of VSD-IV. **a,** VSD-IV voltage-dependent activation in the WT and 11Q mutant as in Fig. 5g. The activation of VSD-IV in WT channel is well approximated by a single Boltzmann distribution (black circles). The neutralization of positive charges in S6^{Cyto} (11Q mutant) allows VSD-IV to visit a new "deep resting" state which is revealed by an increase of the fluorescence signal (unquenching) at

hyperpolarized membrane potentials. Note the biphasic nature of the 11Q $\Delta F(V_m)$ curve (orange squares).

b-c, Cartoon of a structural interpretation of the fluorescence signal from WT and 11Q channels. For simplicity, only S4 helix within VSD-IV is shown to undergo voltage-dependent movements.

b, We speculate that in WT channels with an intact $S6^{Cyto}$, the electrostatic interaction between the VSD-IV and the positive charged residues in $S6^{Cyto}$ (blue) stabilizes this sensor in its resting state disfavoring the occupancy of the deep resting state. In the resting state, the fluorophore is quenched (dim state). Upon membrane depolarization, the S4 segment is displaced causing the conjugated fluorophore to move away from the quencher resulting in an increase in fluorescence as the active state is populated (Fig. 2b, VSD-IV). Thus, in channels with an intact $S6^{Cyto}$, VSD-IV moves between two conformational states (resting and active).

c, 11Q channels lack the electrostatic interaction between positive charges in VSD-IV and $S6^{Cyto}$. VSD-IV can still move between the resting and active states, however the neutralization of the positive charges permits the S4 segment to visit a deep resting state at hyperpolarized potentials. In this state, the fluorophore is unquenched causing a brightening of the fluorescence signal, generating the characteristic biphasic $\Delta F(V_m)$ curve (**a**, 11Q).

a

Channel	Gene Name	Uniprot Accession number	Full-length	VSDs				S4			
				VSD-I (S1-S4)	VSD-II (S1-S4)	VSD-III (S1-S4)	VSD-IV (S1-S4)	VSD-I (S4 only)	VSD-II (S4 only)	VSD-III (S4 only)	VSD-IV (S4 only)
Cav1.1	*CACNA1S*	Q13698	1873	51-183	438-552	794-916	1116-1256	160-182	523-545	888-909	1226-1248
Cav1.2	*CACNA1C*	Q13936-1	2221	124-255	530-644	896-1038	1238-1398	232-254	615-637	1009-1031	1368-1390
Cav1.3	*CACNA1D*	Q01668-1	2161	126-258	529-643	874-1004	1204-1340	235-257	614-636	975-997	1310-1332
Cav1.4	*CACNA1F*	O60840-1	1977	92-224	535-649	859-990	1189-1317	201-223	620-642	960-982	1275-1309
Cav2.1	*CACNA1A*	O00555-8	2506	98-213	492-607	1231-1366	1564-1681	190-212	577-599	1336-1359	1651-1673
Cav2.2	*CACNA1B*	Q00975-1	2339	95-210	489-602	1138-1271	1470-1589	187-209	573-595	1242-1264	1559-1581
Cav2.3	*CACNA1E*	Q15878-1	2313	89-208	482-596	1141-1278	1477-1597	185-207	567-589	1249-1272	1567-1587
Cav3.1	*CACNA1G*	O43497-2	2250	81-199	749-858	1241-1378	1576-1699	175-197	829-851	1349-1372	1666-1691
Cav3.2	*CACNA1H*	O95180-1	2353	100-217	800-909	1282-1419	1616-1739	194-216	879-901	1390-1413	1706-1731
Cav3.3	*CACNA1I*	Q9P0X4-1	2223	79-196	646-755	1158-1295	1485-1609	173-195	726-748	1266-1288	1576-1601
Nav1.1	*SCN1A*	P35498-1	2009	128-234	774-883	1214-1336	1541-1660	211-233	854-876	1307-1329	1627-1652
Nav1.2	*SCN2A*	Q99250-1	2005	129-235	765-874	1204-1326	1531-1650	212-234	845-867	1297-1320	1617-1642
Nav1.3	*SCN3A*	Q9NY46-1	2000	128-234	766-875	1202-1324	1525-1645	211-233	846-868	1295-1318	1612-1637
Nav1.4	*SCN4A*	P35499	1836	131-237	584-693	1027-1149	1352-1472	214-236	664-686	1120-1142	1439-1464
Nav1.5	*SCN5A*	Q14524-1	2016	131-237	723-832	1201-1323	1527-1647	214-236	803-825	1294-1317	1614-1639
Nav1.6	*SCN8A*	Q9UQD0-1	1980	132-238	759-868	1195-1316	1521-1641	215-237	839-861	1287-1310	1607-1633
Nav1.7	*SCN9A*	Q15858-1	1988	126-232	750-859	1188-1310	1514-1634	209-231	830-852	1281-1304	1600-1626
Nav1.8	*SCN10A*	Q9Y5Y9	1956	130-233	671-780	1148-1270	1475-1597	210-232	751-773	12411-263	1563-1589
Nav1.9	*SCN11A*	Q9UI33-1	1791	129-240	583-694	1052-1167	1365-1487	217-239	665-687	1138-1161	1453-1479

b

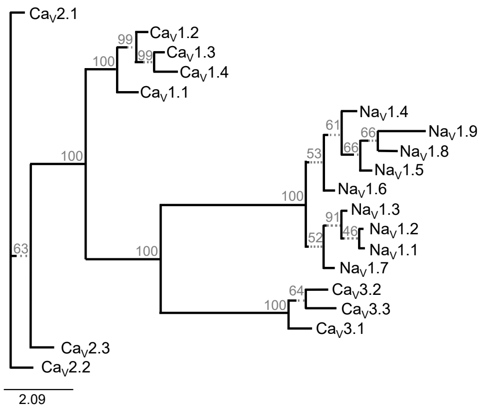

c

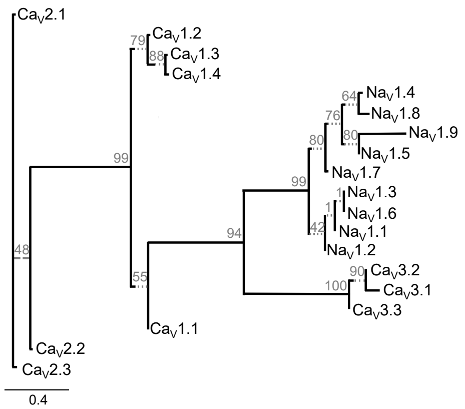

Supplementary Fig. 3: LVA $Ca_V3.1$ and Na_V channels share the same progenitor.

a, The three amino-acid regions used for alignments and tree generation. Table reporting Uniprot accession number of human Ca_V and Na_V channels and amino-acids used for the alignment and the phylogenetic trees.

b-c, Phylogenic trees of Ca_V and Na_V channels obtained from the sequence alignment of the channels full-length **(b)** or VSD S4 region **(c)** using the maximum likelihood approach. Node support values resulting from 1,000 bootstrap replicates are indicated in grey, and scale bar indicates the number of amino acid substitutions per site. All trees, including the one based on VSDs (S1-S4) shown in Fig. 6a, reveal the same common ancestor for Na_V and Ca_V3 channels.

	V_{half} (mV)	z (e_0)	n
**VSD-I**	−50 ± 0.95	4.1 ± 0.14	10
**VSD-II**	−62 ± 1.2	2.3 ± 0.17	7
**VSD-III**	−55 ± 0.84	4.1 ± 0.11	9
**VSD-IV**	−45 ± 0.97	2.7 ± 0.11	8
$G(V_m)$	(1) −37 ± 0.94	(1) 4.2 ± 0.27	13
	(2) 3.4 ± 1.9	(2) 1.4 ± 0.035	
$Q(V_m)$	−2.2 ± 2.1	1.6 ± 0.094	4

Supplementary Table 1: Fitting parameters of the voltage-dependent VSD activation, pore opening and charge movement.

Voltage dependence of VSD activation (fluorescence changes), pore opening and charge movement were fit to a single (VSDs and Q), or the sum of two (G), Boltzmann distributions (Figs. 2 and 4). Values are reported as mean ± SEM. V_{half} is half-activation potential, z is the valence, and n is number of cells. For pore opening, w_1 was 0.61 ± 0.012 and w_2 was 0.39 ± 0.012.

	V_{half} (1) (mV)	*p*	*z* (1) (e_0)	*p*	V_{half} (2) (mV)	*p*	*z* (2) (e_0)	*p*	*n*
**WT**	−37 ± 0.94		4.2 ± 0.27		3.4 ± 1.9		1.4 ± 0.035		13
**S175C (VSD-I)**	−40 ± 0.84	9.11E-02	3.3 ± 0.17	8.00E-03	9.4 ± 1.9	7.24E-02	1.3 ± 0.026	3.14E-01	10
**G829C (VSD-II)**	−40 ± 1.0	1.34E-01	4.0 ± 0.25	9.20E-01	0.12 ± 2.9	6.01E-01	1.4 ± 0.053	1.00E-00	7
**M1353C (VSD-III)**	−37 ± 0.94	1.00E+00	3.8 ± 0.13	4.74E-01	1.1 ± 0.85	8.04E-01	1.4 ± 0.034	9.77E-01	8
**I1669C (VSD-IV)**	−36 ± 0.98	8.00E-01	3.9 ± 0.21	6.94E-01	−2.5 ± 1.4	1.04E-01	1.6 ± 0.033	8.70E-03	8

Supplementary Table 2: Fitting parameters of the voltage-dependent activation of pore conductance in WT and cysteine mutants.

Voltage dependence of pore conductance was fit to the sums of two Boltzmann distributions (Supplementary Fig. 1c). Values are reported as mean ± SEM. WT and cysteine mutants were compared with one-way ANOVA followed with Dunnett's multiple comparisons test. V_{half} is half-activation potential, *z* is the valence, and *n* is number of cells.

		Control	La^{3+}	*p*
**VSD I**	V_{half} (mV)	−50 ± 0.95	−23 ± 1.2	2.35E-09
	z (e_0)	4.1 ± 0.14	2.7 ± 0.10	8.45E-05
	n	10	4	
**VSD II**	V_{half} (mV)	−62 ± 1.2	−68 ± 0.84	2.87E-03
	z (e_0)	2.3 ± 0.17	2.0 ± 0.25	3.92E-01
	n	7	5	
**VSD III**	V_{half} (mV)	−55 ± 0.84	−43 ± 1.3	1.32E-06
	z (e_0)	4.1 ± 0.11	1.8 ± 0.12	1.08E-09
	n	9	7	
**VSD IV**	V_{half} (mV)	−45 ± 0.97	−10 ± 1.3	2.11E-10
	z (e_0)	2.7 ± 0.11	2.2 ± 0.15	1.79E-02
	n	8	5	

Supplementary Table 3: Fitting parameters of the voltage-dependent activation of each VSD in the absence (control) or presence of 200 µM La^{3+}.

Voltage dependence of VSD activation (fluorescence changes) was fit to a Boltzmann distribution (Fig. 4). Values are reported as mean ± SEM, two-tailed unpaired Student's *t*-tests. V_{half} is the half-activation potential, *z* is the valence, and *n* is number of cells.

		WT	11Q	*p*
**VSD I**	V_{half} (mV)	−50 ± 0.95	−41 ± 1.2	6.13E-05
	z (e_0)	4.1 ± 0.14	4.3 ± 0.27	4.37E-01
	n	10	6	
**VSD II**	V_{half} (mV)	−62 ± 1.2	−61 ± 1.5	7.93E-01
	z (e_0)	2.3 ± 0.17	2.1 ± 0.20	5.98E-01
	n	7	4	
**VSD III**	V_{half} (mV)	−55 ± 0.84	−47 ± 1.1	9.38E-05
	z (e_0)	4.1 ± 0.11	4.2 ± 0.25	7.53E-01
	n	9	7	
**VSD IV**	V_{half} (mV)	−45 ± 0.97	−39 ± 1.5	4.36E-03
	z (e_0)	2.7 ± 0.11	4.6 ± 0.35	7.69E-05
	n	8	5	
$G(V_m)$	V_{half} (1) (mV)	−37 ± 0.94	−27 ± 0.85	2.31E-06
	z (1) (e_0)	4.2 ± 0.27	5.8 ± 0.27	2.20E-03
	V_{half} (2) (mV)	3.4 ± 1.9	−3.1 ± 2.8	7.09E-02
	z (2) (e_0)	1.4 ± 0.035	2.0 ± 0.18	1.79E-04
	n	13	6	

Supplementary Table 4: Fitting parameters of the voltage-dependent activation of each VSD in WT or in 11Q mutant.

Voltage dependence of VSD activation (fluorescence changes) and pore opening were fit to a single (VSDs) or the sum of two (*G*) Boltzmann distributions (Fig. 5). Values are reported as mean ± SEM, two-tailed unpaired Student's *t*-tests. V_{half} is half-activation potential, *z* is the valence, and *n* is number of experiments.
