# Supplementary material for: The four voltage-sensing domains of T-type calcium channels activate near the resting membrane potential
Source: Nat Commun. 2026 May 16;17:6506. doi: 10.1038/s41467-026-73077-1 (PMC13377093; doi:10.1038/s41467-026-73077-1)
Supplement: Supplementary file 2 — Reporting Summary [file 41467_2026_73077_MOESM2_ESM.pdf]

nature portfolio

Corresponding author(s): Marina Angelini and Riccardo Olcese

Last updated by author(s): Mar 31, 2026

Reporting Summary

Nature Portfolio wishes to improve the reproducibility of the work that we publish. This form provides structure for consistency and transparency in reporting. For further information on Nature Portfolio policies, see our Editorial Policies and the Editorial Policy Checklist.

Statistics

For all statistical analyses, confirm that the following items are present in the figure legend, table legend, main text, or Methods section.

n/a	Confirmed	
☐	☒	The exact sample size (*n*) for each experimental group/condition, given as a discrete number and unit of measurement
☐	☒	A statement on whether measurements were taken from distinct samples or whether the same sample was measured repeatedly
☐	☒	The statistical test(s) used AND whether they are one- or two-sided<br>*Only common tests should be described solely by name; describe more complex techniques in the Methods section.*
☒	☐	A description of all covariates tested
☒	☐	A description of any assumptions or corrections, such as tests of normality and adjustment for multiple comparisons
☐	☒	A full description of the statistical parameters including central tendency (e.g. means) or other basic estimates (e.g. regression coefficient) AND variation (e.g. standard deviation) or associated estimates of uncertainty (e.g. confidence intervals)
☐	☒	For null hypothesis testing, the test statistic (e.g. *F*, *t*, *r*) with confidence intervals, effect sizes, degrees of freedom and *P* value noted<br>*Give P values as exact values whenever suitable.*
☒	☐	For Bayesian analysis, information on the choice of priors and Markov chain Monte Carlo settings
☒	☐	For hierarchical and complex designs, identification of the appropriate level for tests and full reporting of outcomes
☒	☐	Estimates of effect sizes (e.g. Cohen's *d*, Pearson's *r*), indicating how they were calculated

Our web collection on statistics for biologists contains articles on many of the points above.

Software and code

Policy information about availability of computer code

Data collection	We used acquisition software G-PatchM for current and fluorescence recordings
Data analysis	Analysis software was used to measure and fit current and fluorescence traces; Excel (Micorsoft) and GraphPad Prism 10 were used to fit and plot data and to perform statistical analysis. PyMOL (Schrödinger) was used to visualize structures. NEURON simulation environment was used to simulate neuronal action potentials using the entorhinal cortex layer II stellate neuron model developed by Topczewska et al. (Cell Rep 2023, PMID: 37368752, ModelDB accession no: 266797). Sequence alignment were performed with SeaView 5.0.5 (Gouy et al., Mol Biol Evol. 2010 PMID: 19854763), phylogenetic trees were generated using IQ-TREE (http://iqtree.cibiv.univie.ac.at) and visualized using the ETE Toolkit (https://etetoolkit.org/).

For manuscripts utilizing custom algorithms or software that are central to the research but not yet described in published literature, software must be made available to editors and reviewers. We strongly encourage code deposition in a community repository (e.g. GitHub). See the Nature Portfolio guidelines for submitting code & software for further information.

Data

Policy information about availability of data

All manuscripts must include a data availability statement. This statement should provide the following information, where applicable:

- Accession codes, unique identifiers, or web links for publicly available datasets
- A description of any restrictions on data availability
- For clinical datasets or third party data, please ensure that the statement adheres to our policy

All data are included in the main manuscript and supplemental files. Source data are provided with this paper.
Previously published structures and related PDB codes used in the manuscript are: 6KZO [https://www.wwpdb.org/pdb?id=pdb_00006kzo] and 7WLI [https://www.wwpdb.org/pdb?id=pdb_00007wli].

Research involving human participants, their data, or biological material

Policy information about studies with human participants or human data. See also policy information about sex, gender (identity/presentation), and sexual orientation and race, ethnicity and racism.

Reporting on sex and gender	N/A
Reporting on race, ethnicity, or other socially relevant groupings	N/A
Population characteristics	N/A
Recruitment	N/A
Ethics oversight	N/A

Note that full information on the approval of the study protocol must also be provided in the manuscript.

Field-specific reporting

Please select the one below that is the best fit for your research. If you are not sure, read the appropriate sections before making your selection.

☒ Life sciences ☐ Behavioural & social sciences ☐ Ecological, evolutionary & environmental sciences

For a reference copy of the document with all sections, see nature.com/documents/nr-reporting-summary-flat.pdf

Life sciences study design

All studies must disclose on these points even when the disclosure is negative.

Sample size	Adequate sample size was determined based on published work in the field. A minimum of 4 independent recordings were used for each dataset.
Data exclusions	Oocytes with high leak current, low expression or poor voltage clamp control were excluded from the analysis.
Replication	Experiments from at least 3 independent batches of oocytes were included in each dataset. Additionally, most of the experiment in each dataset were collected from more than one investigator.
Randomization	Oocytes were randomly selected for mRNA injection and randomly selected for experiment.
Blinding	Blinding was not applicable because of the large phenotypically difference among the mutants used in the study.<br>In the type of experiments presented the operators cannot influence the outcome of the measurement.

Reporting for specific materials, systems and methods

We require information from authors about some types of materials, experimental systems and methods used in many studies. Here, indicate whether each material, system or method listed is relevant to your study. If you are not sure if a list item applies to your research, read the appropriate section before selecting a response.

Materials & experimental systems

n/a	Involved in the study
☒	☐ Antibodies
☒	☐ Eukaryotic cell lines
☒	☐ Palaeontology and archaeology
☐	☒ Animals and other organisms
☒	☐ Clinical data
☒	☐ Dual use research of concern
☒	☐ Plants

Methods

n/a	Involved in the study
☒	☐ ChIP-seq
☒	☐ Flow cytometry
☒	☐ MRI-based neuroimaging

Animals and other research organisms

Policy information about studies involving animals; ARRIVE guidelines recommended for reporting animal research, and Sex and Gender in Research

Laboratory animals	Female Xenopus laevis frogs were used to harvest oocytes.
Wild animals	This study did not involve wild animals.
Reporting on sex	Female
Field-collected samples	The study did not involved samples collected from the field.
Ethics oversight	All animal protocols were approved by the UCLA Institutional Animal Care and Use Committee and conformed to the Guide for the Care and Use of Laboratory Animals published by the U.S. National Institutes of Health.

Note that full information on the approval of the study protocol must also be provided in the manuscript.

Plants

Seed stocks	N/A
Novel plant genotypes	N/A
Authentication	N/A
