# Supplementary material for: The four voltage-sensing domains of T-type calcium channels activate near the resting membrane potential
Source: Nat Commun. 2026 May 16;17:6506. doi: 10.1038/s41467-026-73077-1 (PMC13377093; doi:10.1038/s41467-026-73077-1)
Supplement: Supplementary file 3 — Transparent Peer Review file [file 41467_2026_73077_MOESM3_ESM.pdf]

nature portfolio

Peer Review File

The Four Voltage-Sensing Domains of T-Type Calcium Channels Activate Near the Resting Membrane Potential

Corresponding Author: Professor Riccardo Olcese

Parts of this Peer Review File have been redacted as indicated to maintain the confidentiality of unpublished data.

This file contains all reviewer reports in order by version, followed by all author rebuttals in order by version.

Version 0:

Reviewer comments:

Reviewer #1

(Remarks to the Author)
Agelini et al., studied the voltage-dependent activity of CaV3.1 VSDs using voltage-clamp fluorometry (VCF). CaV3/LVA channels activate at voltages close the neuronal resting membrane potential, allowing them to regulate cellular excitability and pace-making currents. Earlier studies that measured gating currents (with extracellular La3+ block) of LVA channels paradoxically found that the VSDs move/activate at more depolarized voltages than the activation of calcium influx. Here, Angelini et al. track the activation/movement of each of the four individual VSDs of CaV3.1 by using the VCF technique. They found that each of the four VSDs activate at hyperpolarized potentials, all at lower voltages than the opening of the pore (G(V)). However, in the presence of La3+, the voltage dependence of VSD movement shifted to the right, explaining the paradoxical findings of the past. Additionally, this study tests the importance of the positively charged cytosolic extension of IIIS6 (S6cyto) for CaV3.1 gating, previously found to contribute CaV3.3 LVA properties. By neutralizing S6cyto, both pore opening and VSD movement shifted significantly to more depolarized potentials, corroborating the importance of S6cyto for LVA properties of CaV3 channels and demonstrating the involvement of the VSDs in the S6cyto effect. Remarkably, VSD II remains unaffected by S6cyto neutralization, suggesting a lesser contribution or uncoupling to pore opening for this VSD. Finally, the authors use the VCF data from this and previous VCF studies, in combination with sequence alignments, to draw conclusions on evolution/phylogeny.

This study characterizes the unique properties of CaV3.1 VSDs (representative of T-type channels), and shows for the first time that all four VSDs of CaV3.1 activate within a narrow voltage window, preceding the opening of the pore. The paradoxical results from earlier gating-charge measurements are explained by the observed right-shifted VSD activation accompanying pore block by La3+. This solves a longstanding conundrum in the field and offers highly valuable insights into LVA VSD activation. Another interesting revelation is that the individual VSDs of LVA channels all activate in the same narrow voltage-window, in contrast to previously observed high variation among the individual VSDs of HVA channels.

Moreover, this study shows that one of the structural underpinnings of the LVA properties of CaV3.1 VSDs is the S6cyto. Agelini et al. suggest an intriguing structural model (testable hypothesis) proposing that repulsion of IVS4 by the positively charged S6cyto prevents a “deep-resting” state, thus facilitating the transition into the activated state and activation at low-voltage. Finally, the structure-function comparison of VCF data from CaV and NaV channels indicates a correlation of heterogeneity in VSD function and gating properties. This is interesting but the evolutional interpretation is purely speculative (see below).

Experimentally, the study is of high quality and the data are presented clearly and convincingly. Whereas we do have some serious criticism, this first VCF study of a T-type channel is a valuable contribution to the field containing a number of interesting findings, worth being published in Nature Communication.

Major:

1. The n numbers of experiments are only given in the Supplementary tables and nowhere in the main text. This is insufficient. The n numbers and statistical tests used for each experiment should be mentioned in the figures and/or the

figure legends.

2. Fig. 3e. Displaying sample recordings of different command potentials in the same lines is confusing and does not allow comparing the VSD movements at identical potentials. All lines need to have the same command voltage, as shown in Fig. 2 (even if that means to add additional lines of traces to this figure).

3. Statistical test should be shown to demonstrate that there is no difference between the Vhalf of WT and the tagged constructs.

4. In the title and in the final chapter of the Discussion the authors evoke evolution. The title, “The Voltage Sensing Domains of T-Type Calcium Channels Evolved to Activate Near the Cell Membrane Resting Potential” poorly represents the essence of the study, and needs to be changed. In fact, the study does not at all address evolution experimentally, nor does the sequence comparison of human NaV and CaV channels represent a phylogenetic tree. To “examine the evolutionary hypothesis” (line 329) or to conclude about ancestry (line 334), channels from species across the phyla need to be compared. In light of this, the title is misleading and the evolutionary interpretation of the data is far-fetched and inappropriate.

5. Instead, a thorough discussion of possible structure-function correlations would be indicated and of much greater value. For example, which structural similarities could explain the more homogenous functions of VSDs in NaVs and CaV3´s compared to the HVA CaVs? Or, what are the structural similarities in VSD II´s from various channels, which render them poorly coupled to the pore or locked in the resting states?

6. The authors stress the homogenous behavior of the four VSDs in terms of voltage-dependence of activation. However, they do not analyze or discuss the obvious differences in kinetics. In their previous work VSD kinetics was considered an important characteristics to identify the contribution of specific VSDs in channel activation or depolarization-induced calcium release in skeletal muscle. It would be interesting to compare activation kinetics of CaV3.1 with kinetics of current activation. For example, VSD I activates very slowly. Is this compatible with a substantial contribution to pore opening? Can the quantitative allosteric model, developed for CaV1.2 by the group, be applied to CaV3.1?

7. The paragraph on the HCN channels (Discussion, 238-252) is out of place in this article. It does not relate to the presented experiments and does not offer substantial new insight. It better be removed entirely.

8. Line 46 “all other CaV channels, which only activate in response to full depolarization”. This does not apply for CaV1.3. Therefore, “all” should be avoided in this context. More importantly, the statement in the Discussion (line 326; “a feature among all HVA channels”) relates only to all examined HVA channels. Without VCF data on CaV1.3 this claim cannot be held. Let alone that scientific theory maintains that claims about “all” of something require that every possible case has been studied. A comparison of 5/10 CaVs and 2/10 NaVs (without considering splice variants) is valuable and indicative, but insufficient to draw such general conclusions.

Minor:
1. The source of the sigmodal Bolzman curves in Fig 1a should be cited in the legend.
2. In legend of Fig 1c indicate that black circles are calcium ions.
3. In line 154, why only give approximate Vhalf of G(V) but precise average and SEM for Q(V)?
4. Line 341-342 “T-type CaV is the progenitor for all the subsequence NaV channels.” Wasn’t the point that they have a direct common ancestor, not that NaV comes from CaV3?

Orthographical:
1. Line 48 “referred-to”, remove hyphen.
2. Sentence in line 53 and 54 would be better like this: “…, the origin of the low-voltage-sensitivity of LVA channels remains…”
3. Line 56 has double “of”: “..architecture of typical of voltage-gated channels..”
4. Line 71-71 remove hyphen in voltage-dependence (check for consistency throughout the article).
5. Line 97 remove comma after thiol-reactive.
6. Line 105-106, should refer to Fig 2b, not 2a.
7. Line 568 legend Fig 2 change to voltage-step protocols.
8. Line 188, revealed not reveal.
9. Line 243 “…so that the pore opens as the VSDs…”, plural “s”
10. Line 257 “specifically VSDs voltage dependence”; do you mean “the voltage dependence of specific VSDs”?.
11. Line 285, what is meant with “all” VSD-IV?
12. Line 631 (Fig 5) VSD or VSD’s not VSDs.

Reviewer #2

(Remarks to the Author)
I co-reviewed this manuscript with one of the reviewers who provided the listed reports. This is part of the Nature Communications initiative to facilitate training in peer review and to provide appropriate recognition for Early Career Researchers who co-review manuscripts.

Reviewer #3

(Remarks to the Author)
This study by Angelini et al. addresses a fundamentally significant question in ion channel biophysics: resolving the long-standing paradox of LVA in T-type Cav3 channels. The authors employ rigorous methodologies, including voltage-clamp fluorometry (VCF) and mutagenesis, to dissect VSD dynamics and identify confounding factors. The work is technically sophisticated and provides valuable insights into Cav3 gating mechanisms. However, while the data are robust for observational conclusions, they fall short of supporting the mechanistic and evolutionary claims made in the manuscript. Below, I outline key strengths followed by critical concerns.
Although numerous studies indicate that pore opening in voltage-gated channels does not require activation of all VSDs, cooperative interactions among VSDs are critical for channel activation. The authors demonstrate that all four VSDs of the Cav3.1 channel activate near the resting membrane potential, with their activation voltages clustered within a narrow range (~15 mV). Does the narrow voltage range of VSD activation imply strong cooperativity, enabling low-voltage pore opening in Cav3.1 channels?
Why did the study choose 200 μM La^{3+}? The authors show that La^{3+} (used to block ionic currents in gating experiments) artificially shifts VSD activation toward depolarized potentials (Fig. 3). This explains historical discrepancies and underscores VCF's utility for studying intact channels. However, it remains unclear whether La^{3+} directly interacts with VSDs to alter their voltage dependence. Why not use small-molecule pore blockers? For some, structures with Cav3.1 have been resolved (PMID: 31766050), with clearly defined binding sites.
Building on the work of He et al. (2022, PMID: 35440630), who used structural and mutagenesis approaches to highlight the critical role of the positively charged Domain III S6cyto loop in low-voltage activation gating, this study employs VCF to demonstrate S6Cyto's influence on VSD activation (particularly VSD-IV). The authors propose that the positively charged S6Cyto electrostatically counteracts the VSD-IV deep resting state, thereby minimizing the energy required for activation and facilitating channel opening. However, direct mechanistic evidence supporting this model is lacking in the manuscript.
The authors suggest that "the CaV3.1 DII VSD is unlikely to contribute to pore opening". However, a study found that a spider toxin selectively targeting the DII VSD potently inhibits Cav3.1 current and impedes channel opening (PMID: 35858123), which indirectly suggests that the DII VSD is critical for pore opening.
Overinterpretation of evolutionary claims：The assertion that "Nav-like VSDs emerged before sodium selectivity" is intriguing but remains speculative, and they provide no direct evidence for this sequence of events. The phylogenetic tree (Fig. 5a) lacks bootstrap values, weakening its robustness. Perform bootstrap analysis (1,000 replicates) to support the Cav3/Nav clade. In addition, the analysis includes only human channel sequences (19 total). This limited scope ignores potential insights from basal organisms (e.g., invertebrates or early vertebrates), which could provide stronger tests of evolutionary divergence. Without broader taxonomic representation, the tree may not accurately reflect deep evolutionary relationships.
According to the authors' phylogenetic tree analysis, Cav3 channels share a closer evolutionary relationship with Nav channel subtypes Nav1.8 and Nav1.9. However, Nav1.8 and Nav1.9 exhibit stark differences in activation kinetics: Nav1.9 opens near resting membrane potentials (low-threshold activation), similar to LVA Cav3 channels.Nav1.8 has the highest activation threshold among Nav channels, resembling HVACav channels. How can this be reconciled?"
The authors claim that Cav3.1 channels share high similarity in voltage-dependent properties with Nav1.4 and Nav1.5. However, Fig.5 shows that Nav1.4 VSDs activate at lower voltages than Cav3.1 VSDs. According to the authors' theory, Nav1.4 pore opening should therefore occur at lower voltages than Cav3.1. Yet in the figure, Nav1.4 pore opening occurs around -40 mV, while Cav3.1 opens near -60 mV. Why?
Specify the alignment algorithm and tree-building method (e.g., maximum-likelihood) in Methods.

Version 1:

Reviewer comments:

Reviewer #1

(Remarks to the Author)
In the revised version of their manuscript, Angelini et al. have satisfied the great majority of my previous comments and criticisms and I consider the article suitable for publication.

Most importantly, they have completely redrawn figure 3 and added the analysis of activation kinetics. This analysis demonstrates that activation of all four VSDs contains a component sufficiently fast to account for the rapid kinetics of pore opening. This additional data substantially improved the study. Of course, the observation that the bulk of the movement of VSDs I and IV (slow component) occurs after pore opening merits a brief explanatory statement. Do these VSDs have multiple activated states or is their partial S4 transition sufficient to activate pore opening?

The authors also have removed the rather misleading reference to evolution from the title. However other than this, they chose to adhere to their interpretation of the data in terms of channel evolution. While their sequence analysis adds valuable new evidence to this discussion, the contribution of their functional analysis to this debate is questionable. Considering the existing VCF data shown in figure 6, the statement, "By comparing the VSDs of CaV1 12-14,17, CaV2 15,16, CaV3 (this work) and NaV channels 49,50 (Fig. 6), we provide evidence that the CaV3.1 VSDs operate more similarly to those of a NaV channels than HVA CaV-channels, in terms of overall voltage sensitivity and range of half-activation potentials (Fig. 6)." lacks sufficient experimental support. The authors compare 1 of 3 CaV3 channels with 2 of 10 NaV channels. Indeed, the

narrow range of activation voltages of CaV3.1 is similar to that of NaV1.4; however, its voltage sensitivity or voltage-dependence of activation are not at all similar. Furthermore, the narrow range of CaV3.1 VSD activation is dissimilar to that of the other example NaV1.5 (the activation voltages of which ranges over 50 mV and thus is much more similar to that of CaV1 channels). At this point, we do not know the properties of the other two CaV3 channels, neither do we know whether the other eight NaV channels exhibit properties more like NaV1.4 or NaV1.5, or show even greater differences to those of CaV3.1. Consequently, the current study adds one important data point to this question, but is far from demonstrating that, "… our VCF data support experimentally the CaV3.1 evolutionary vicinity to NaV-channels and their distinction from HVA CaV channels."

Having said this, I want to re-emphasize the importance and excellent quality of this VCF study and that I am looking forward to seeing it published in Nature Communications.

Reviewer #2

(Remarks to the Author)
I co-reviewed this manuscript with one of the reviewers who provided the listed reports. This is part of the Nature Communications initiative to facilitate training in peer review and to provide appropriate recognition for Early Career Researchers who co-review manuscripts.

Reviewer #3

(Remarks to the Author)
Thank you for your detailed responses to the previous comments. Regarding point 2, the authors state that the Cav3 channel-specific inhibitor Z944 was used. However, no corresponding data is presented in the response to support this crucial methodological claim.

Version 2:

Reviewer comments:

Reviewer #1

(Remarks to the Author)
With the additional clarifications and by toning down their conclusions on the evolution of Cav and Nav channels, the authors have satisfied all my previous concerns. I consider the article suitable for publication in Nature Communications.

Reviewer #2

(Remarks to the Author)
I co-reviewed this manuscript with one of the reviewers who provided the listed reports. This is part of the Nature Communications initiative to facilitate training in peer review and to provide appropriate recognition for Early Career Researchers who co-review manuscripts.

Reviewer #3

(Remarks to the Author)
I have no further suggestions.

Open Access This Peer Review File is licensed under a Creative Commons Attribution 4.0 International License, which permits use, sharing, adaptation, distribution and reproduction in any medium or format, as long as you give appropriate credit to the original author(s) and the source, provide a link to the Creative Commons license, and indicate if changes were made.
In cases where reviewers are anonymous, credit should be given to 'Anonymous Referee' and the source.
The images or other third party material in this Peer Review File are included in the article's Creative Commons license, unless indicated otherwise in a credit line to the material. If material is not included in the article's Creative Commons license and your intended use is not permitted by statutory regulation or exceeds the permitted use, you will need to obtain permission directly from the copyright holder.
To view a copy of this license, visit https://creativecommons.org/licenses/by/4.0/

NCOMMS-25-53611-A RESPONSE TO REVIEWER COMMENTS

We sincerely thank the Reviewers for the positive appraisal of our work and constructive comments. We have addressed all the concerns and made changes to the revised manuscript accordingly.

We have conducted additional analysis and included a new Figure 3 describing the kinetic of activation of individual VSDs and modified Figure 3, 5 (Figure 4 and 6 in the revised manuscript) and Supplementary Figure 3, as suggested by the Reviewers. We have included a co-author, Dr. Andreas Schwingshackl (UCLA).

Major changes in the manuscript are in blue color fonts.

Please find below our point-by-point answers to Reviewers' comments quoted in blue font, Italic.

Reviewer #1 (Remarks to the Author):

Agelini et al., studied the voltage-dependent activity of CaV3.1 VSDs using voltage-clamp fluorometry (VCF). CaV3/LVA channels activate at voltages close the neuronal resting membrane potential, allowing them to regulate cellular excitability and pace-making currents. Earlier studies that measured gating currents (with extracellular La3+ block) of LVA channels paradoxically found that the VSDs move/activate at more depolarized voltages than the activation of calcium influx. Here, Angelini et al. track the activation/movement of each of the four individual VSDs of CaV3.1 by using the VCF technique. They found that each of the four VSDs activate at hyperpolarized potentials, all at lower voltages than the opening of the pore (G(V)). However, in the presence of La3+, the voltage dependence of VSD movement shifted to the right, explaining the paradoxical findings of the past. Additionally, this study tests the importance of the positively charged cytosolic extension of IIIS6 (S6cyto) for CaV3.1 gating, previously found to contribute CaV3.3 LVA properties. By neutralizing S6cyto, both pore opening and VSD movement shifted significantly to more depolarized potentials, corroborating the importance of S6cyto for LVA properties of CaV3 channels and demonstrating the involvement of the VSDs in the S6cyto effect. Remarkably, VSD II remains unaffected by S6cyto neutralization, suggesting a lesser contribution or uncoupling to pore opening for this VSD. Finally, the authors use the VCF data from this and previous VCF studies, in combination with sequence alignments, to draw conclusions on evolution/phylogeny.

This study characterizes the unique properties of CaV3.1 VSDs (representative of T-type channels), and shows for the first time that all four VSDs of CaV3.1 activate within a narrow voltage window, preceding the opening of the pore. The paradoxical results from earlier gating-charge measurements are explained by the observed right-shifted VSD activation accompanying pore block by La3+. This solves a longstanding conundrum in

the field and offers highly valuable insights into LVA VSD activation. Another interesting revelation is that the individual VSDs of LVA channels all activate in the same narrow voltage-window, in contrast to previously observed high variation among the individual VSDs of HVA channels.

Moreover, this study shows that one of the structural underpinnings of the LVA properties of CaV3.1 VSDs is the S6cyto. Agelini et al. suggest an intriguing structural model (testable hypothesis) proposing that repulsion of IVS4 by the positively charged S6cyto prevents a "deep-resting" state, thus facilitating the transition into the activated state and activation at low-voltage. Finally, the structure-function comparison of VCF data from CaV and NaV channels indicates a correlation of heterogeneity in VSD function and gating properties. This is interesting but the evolutional interpretation is purely speculative (see below).

Experimentally, the study is of high quality and the data are presented clearly and convincingly. Whereas we do have some serious criticism, this first VCF study of a T-type channel is a valuable contribution to the field containing a number of interesting findings, worth being published in Nature Communication.

We thank the Reviewer for the positive assessment of our study. We have addressed all the concerns and made changes to the manuscript accordingly.

Major:

1. The n numbers of experiments are only given in the Supplementary tables and nowhere in the main text. This is insufficient. The n numbers and statistical tests used for each experiment should be mentioned in the figures and/or the figure legends.

We apologize for the oversight. We have added the *n* numbers and statistical test used in the figure legends as suggested by the Reviewer.

2. Fig. 3e. Displaying sample recordings of different command potentials in the same lines is confusing and does not allow comparing the VSD movements at identical potentials. All lines need to have the same command voltage, as shown in Fig. 2 (even if that means to add additional lines of traces to this figure).

We thank the Reviewer for the suggestion. Accordingly, we have modified Figure 3 (Figure 4 in the revised manuscript) to show VSD movements at the same membrane potentials.

3. Statistical test should be shown to demonstrate that there is no difference between the Vhalf of WT and the tagged constructs.

We agree and have performed statistical analyses to compare the V_{half} values of WT $Ca_V3.1$ and tagged constructs: the *p* values are provided in Supplementary Table 2. Differences between the WT clone and each individual cysteine mutant were assessed

using a one-way analysis of variance (ANOVA) followed by Dunnett's multiple comparisons test. None of the tagged constructs exhibited V_{half} activation values that were statistically different from the WT. A small but significant change in the valence (z) of one component of the double Boltzmann fit was observed for cysteine mutants in Repeats I (z_1 with p = 8.00E−03) and IV (z_2 with p = 8.70E−03). Overall, the cysteine mutants used in this study retain WT-like properties.

We have modified the Result as follows (page 4, lines 123-128):

"Overall, the $G(V_m)$ of labeled channels carrying Cys mutations for VCF closely recapitulate the functional properties of WT channels (Supplementary Fig. 1c and Supplementary Table 2). Although a small difference in the valence (z) of one component of the double Boltzmann fit was observed for Cys mutants in Repeats I and IV (Supplementary Fig. 1c and Supplementary Table 2), the Cys mutants used in this study largely retain WT-like properties."

We have modified the Methods to include the statistical test used (page 12, lines 469-471):

"*p* values were calculated using two-tailed unpaired Student's *t*-tests or one-way analysis of variance (ANOVA) followed by Dunnett's multiple comparisons."

4. In the title and in the final chapter of the Discussion the authors evoke evolution. The title, "The Voltage Sensing Domains of T-Type Calcium Channels Evolved to Activate Near the Cell Membrane Resting Potential" poorly represents the essence of the study, and needs to be changed. In fact, the study does not at all address evolution experimentally, nor does the sequence comparison of human NaV and CaV channels represent a phylogenetic tree. To "examine the evolutionary hypothesis" (line 329) or to conclude about ancestry (line 334), channels from species across the phyla need to be compared. In light of this, the title is misleading and the evolutionary interpretation of the data is far-fetched and inappropriate. Instead, a thorough discussion of possible structure-function correlations would be indicated and of much greater value. For example, which structural similarities could explain the more homogenous functions of VSDs in NaVs and CaV3´s compared to the HVA CaVs? Or, what are the structural similarities in VSD II´s from various channels, which render them poorly coupled to the pore or locked in the resting states?

We agree with the Reviewer, and we have modified the title as follows: "*The Four Voltage-Sensing Domains of T-Type Calcium Channels Activate Near the Resting Membrane Potential*".

Regarding the Reviewer's comments about evolution, we agree that our study does not address evolutionary relationships across species by directly comparing Ca_V and Na_V channels from different organisms. However, by comparing results from various VCF

studies of Ca_V and Na_V channels, we found intriguing functional commonalities pertaining to the voltage dependence of their VSDs. We believe these similarities warrant discussion, especially in view of the evolutionary theories proposed by others. Therefore, we have purposely assigned this part to the Discussion of the manuscript.

In Figure 6 of the revised manuscript presented in the Discussion, we have overlaid fundamental properties of the VSDs upon a phylogenetic tree derived from all human Ca_V and Na_V channel amino acid sequences. This exercise emphasizes that $Ca_V3.1$ channels are more closely related to Na_V channels than to the other HVA Ca_V channels. All four VSDs of $Ca_V3.1$ display prominent hyperpolarized activation V_{half} values consistent with properties of low-voltage-activated channels.

Thus, we regard the overlap of the VCF data and phylogenetic tree as supportive of the general trend the tree provided, implying that Na_V channels derived from a common Ca_V ancestor: in our hands we produce the gating features and sequence analysis to suggest that the ancestor was likely a Ca_V2 channel homolog. Further functional and sequence study of channels from different species in the future will help in more narrowly defining this trend.

Altogether, we believe that this point in the Discussion section, based on functional data and sequence comparisons, provides support to a current evolutionary theory and offers a starting point for future investigations.

We do take this Reviewer's point and have accordingly added a statement to the Discussion (page 9, lines 340-345):

"The overlap of the VSD functional and sequence analyses (Fig. 6) enable us to lend experimental support to the theory that T-type Ca_V and Na_V share a common ancestor, as proposed 20 years ago. Such analysis has the implication that low-voltage-activating VSDs emerged in LVA Ca_V-channels prior to the Ca^{2+} to Na^+ switch in ionic selectivity. The evolutionary advance of this change, which allowed separation of electrical excitation and Ca^{2+} signaling, is an intriguing area of future study."

Regarding the suggestion of discussing similarities between Na_V and Ca_V channels we were unable to identify any obvious structural similarities that clearly distinguish Na_V/Ca_V3 channels from HVA Ca_V channels; therefore, we have refrained from including this discussion in the manuscript.

5. The authors stress the homogenous behavior of the four VSDs in terms of voltage-dependence of activation. However, they do not analyze or discuss the obvious differences in kinetics. In their previous work VSD kinetics was considered an important characteristics to identify the contribution of specific VSDs in channel activation or depolarization-induced calcium release in skeletal muscle. It would be interesting to compare activation kinetics of CaV3.1 with kinetics of current activation. For example,

VSD I activates very slowly. Is this compatible with a substantial contribution to pore opening? Can the quantitative allosteric model, developed for CaV1.2 by the group, be applied to CaV3.1?

We appreciate your insightful comment. We have analyzed the time course of VSD activations by fitting fluorescence recordings to single or double exponential functions and compared the VSD activation with the kinetics of current activation. Overall, we observed that all VSDs display at least a fast component in the activation that is compatible with the activation kinetic of the ionic current. Based on this new analysis, all VSDs possess biophysical properties (activation time course and voltage dependence) compatible with a role in channel activation. We have added a new Figure 3 (see below) and modified the manuscript accordingly.

Certainly, an exhaustive computational study aimed at developing an entirely new model of CaV3.1 activation (incorporating experimental constraints for the time- and voltage-dependent properties of the four VSDs and the pore) would be ideal. However, such a modeling exercise warrants full, dedicated study that, while in progress, is beyond the scope of the present study in which we describe for the first time the activation of VSDs in a Low-voltage gated CaV channel.

New Figure 3

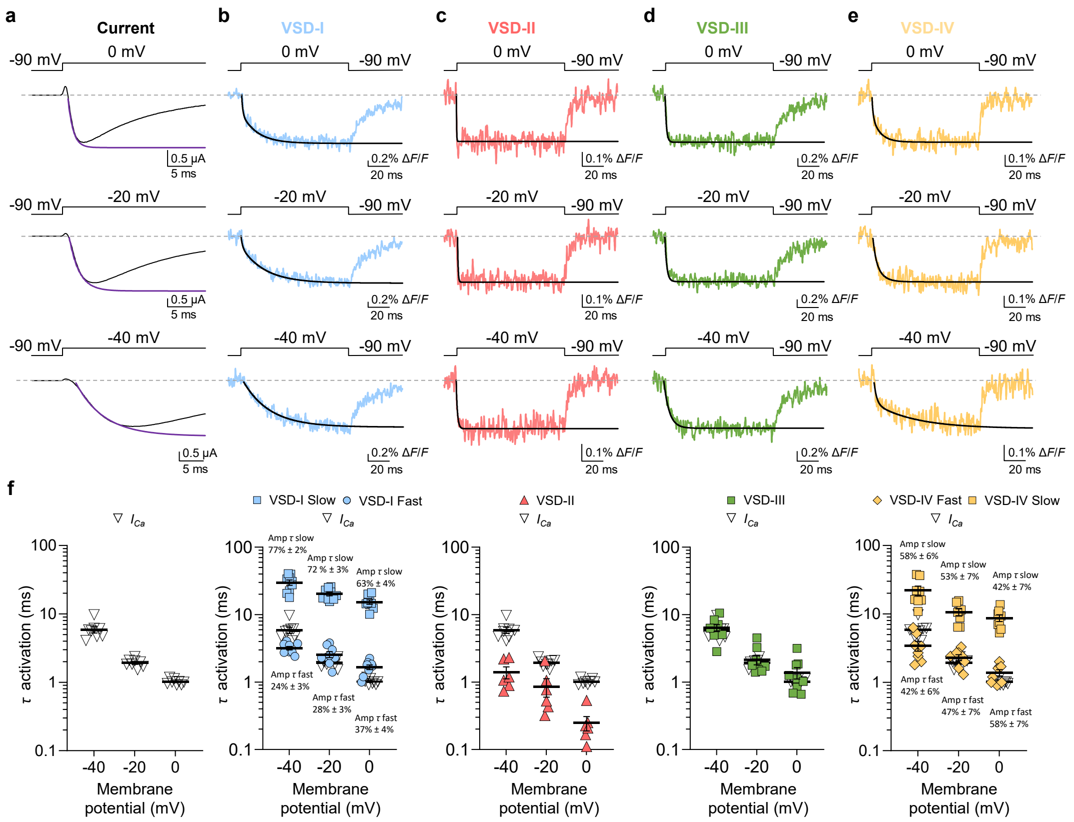

Figure 3: The activation kinetics of all VSDs are compatible with the pore opening.
a, Representative WT CaV3.1 ionic current recordings (black) elicited by depolarizations to −40 mV, −20 mV and 0 mV. Superimposed purple lines are the best fits to an exponential function describing ionic current activation.
b-e, Representative fluorescence traces of the four VSDs (in colors). The activation kinetics of each VSD were well described by a single (VSD-II and -III) or double (VSD-I and VSD-IV) exponential function (superimposed black lines).
f, The time constants (τ) of ionic current onset (black open triangles) and each VSD activation (colored symbols) at the indicated membrane potentials. For VSD-I and VSD-IV, the relative amplitudes (Amp%) of fast and slow components are reported above and below the respective τ values. Note that all VSDs exhibit time course of activation equal or faster to that of ionic current onset. (Current: *n* = 8, VSD-I: *n* = 9, VSD-II: *n* = 6, VSD-III: *n* = 9, VSD-IV: *n* = 8).

We have modified the text and included the newly performed analysis in Result (page 4-5, lines 141-150) as follows:

"Each VSD reacted to depolarizing pulses with distinct kinetics (Fig. 3). VSD-II and VSD-III activated very rapidly with time constants (τ) faster or equal to those of the pore opening (at −20 mV, ionic current: τ = 1.9 ± 0.091 ms; VSD-II: τ = 0.86 ± 0.26 ms; VSD-III: τ = 2.1 ± 0.35 ms; Fig. 3a, c, d, f). In contrast, VSD-I and VSD-IV activations were well fit by the sum of two exponential functions. The fast components of VSD-I (at −20mV, τ_{fast} = 2.6 ± 0.26 ms, accounting for 28% ± 3% of total amplitude) and VSD-IV (τ_{fast} = 2.3 ± 0.23 ms, 47% ± 7% of total amplitude) were also comparable to the rate of channel opening (Fig. 3b, e, f).

Altogether these data supported the premise that the low-voltage dependence of low-voltage-activated channels emerges from the low-voltage-dependent activation of its four distinct VSDs."

6. The paragraph on the HCN channels (Discussion, 238-252) is out of place in this article. It does not relate to the presented experiments and does not offer substantial new insight. It better be removed entirely.

We agree with the Reviewer that the paragraph discussing HCN channel did not provide new insights. We have removed that paragraph from the Discussion.

7. Line 46 "all other CaV channels, which only activate in response to full depolarization". This does not apply for CaV1.3. Therefore, "all" should be avoided in this context. More importantly, the statement in the Discussion (line 326; "a feature among all HVA channels") relates only to all examined HVA channels. Without VCF data on CaV1.3 this claim cannot be held. Let alone that scientific theory maintains that claims about "all" of something require that every possible case has been studied. A comparison of 5/10 CaVs

and 2/10 NaVs (without considering splice variants) is valuable and indicative, but insufficient to draw such general conclusions.

We agree with the Reviewer, and we apologize for the lack of precision. We have now removed the word "all" and modified the Discussion accordingly, as not all the HVA channels have been examined using VCF.

Minor:

1. The source of the sigmodal Bolzman curves in Fig 1a should be cited in the legend.

Thank you, we have included the references in the Figure1 legend.

2. In legend of Fig 1c indicate that black circles are calcium ions.

Done.

3. In line 154, why only give approximate Vhalf of G(V) but precise average and SEM for Q(V)?

We now report the average V_{half} of $G(V)$ with SEM estimated from the fit.

4. Line 341-342 "T-type CaV is the progenitor for all the subsequence NaV channels." Wasn't the point that they have a direct common ancestor, not that NaV comes from CaV3?

The Reviewer is correct, and we have corrected the text (Page 9, Lines 340-341) as follows:

"The overlap of the VSD functional and sequence analyses (Fig. 6) enable us to lend experimental support to the theory that T-type Ca_V and Na_V share a common ancestor, as proposed 20 years ago."

Orthographical:

1. Line 48 "referred-to", remove hyphen.

Done.

2. Sentence in line 53 and 54 would be better like this: "…, the origin of the low-voltage-sensitivity of LVA channels remains…"

Done.

3. Line 56 has double "of": "..architecture of typical of voltage-gated channels.."

Done.

4. Line 71-71 remove hyphen in voltage-dependence (check for consistency throughout the article).

Done.

5. Line 97 remove comma after thiol-reactive.

Done.

6. Line 105-106, should refer to Fig 2b, not 2a.

Done.

7. Line 568 legend Fig 2 change to voltage-step protocols.

Done.

8. Line 188, revealed not reveal.

Done.

9. Line 243 "…so that the pore opens as the VSDs…", plural "s"

Done.

10. Line 257 "specifically VSDs voltage dependence"; do you mean "the voltage dependence of specific VSDs"?.

Done. We have changed the sentence as suggested by the Reviewer.

11. Line 285, what is meant with "all" VSD-IV?

We have removed the word "all".

12. Line 631 (Fig 5) VSD or VSD's not VSDs.

Done. We have modified this with VSD.

Reviewer #2 (Remarks to the Author):

I co-reviewed this manuscript with one of the reviewers who provided the listed reports. This is part of the Nature Communications initiative to facilitate training in peer review and to provide appropriate recognition for Early Career Researchers who co-review manuscripts.

Thank you for co-reviewing our manuscript!

Reviewer #3 (Remarks to the Author):

This study by Angelini et al. addresses a fundamentally significant question in ion channel biophysics: resolving the long-standing paradox of LVA in T-type Cav3 channels. The

authors employ rigorous methodologies, including voltage-clamp fluorometry (VCF) and mutagenesis, to dissect VSD dynamics and identify confounding factors. The work is technically sophisticated and provides valuable insights into Cav3 gating mechanisms. However, while the data are robust for observational conclusions, they fall short of supporting the mechanistic and evolutionary claims made in the manuscript. Below, I outline key strengths followed by critical concerns.

We thank the Reviewer for the positive appraisal of our study. We have addressed all the concerns and made changes to the manuscript accordingly.

1-Although numerous studies indicate that pore opening in voltage-gated channels does not require activation of all VSDs, cooperative interactions among VSDs are critical for channel activation. The authors demonstrate that all four VSDs of the Cav3.1 channel activate near the resting membrane potential, with their activation voltages clustered within a narrow range (~15 mV). Does the narrow voltage range of VSD activation imply strong cooperativity, enabling low-voltage pore opening in Cav3.1 channels?

We appreciate the Reviewer's insightful comment. The narrow voltage range of VSD activation in $Ca_V3.1$ is consistent with some degree of cooperativity among voltage sensors, as demonstrated for $Na_V1.4$ (Chanda et al., *JGP* 2004 PMID: 14981134). In principle, (and without involving direct cooperativity among VSDs) the central pore serves as an allosteric center for the VSDs that are energetically coupled to it. Thus, a perturbation of any VSD energetically coupled to the pore should reflect back to other VSDs that are also strongly energetically coupled to pore opening. However, we have not yet systematically investigated potential cooperativity among the $Ca_V3.1$ VSDs, either experimentally or through kinetic modeling.

2-Why did the study choose 200 μM La^{3+}? The authors show that La^{3+} (used to block ionic currents in gating experiments) artificially shifts VSD activation toward depolarized potentials (Fig. 3). This explains historical discrepancies and underscores VCF's utility for studying intact channels. However, it remains unclear whether La^{3+} directly interacts with VSDs to alter their voltage dependence. Why not use small-molecule pore blockers? For some, structures with Cav3.1 have been resolved (PMID: 31766050), with clearly defined binding sites.

Thank you for the opportunity to clarify this point. In the COVG-VCF experiments we used 200 μM La^{3+} (rather than 1mM as in the gating current experiments) to minimize potential surface charge screening effects that can occur in the presence of trivalent cations while still achieving potent ionic current blockade.

As discussed in the manuscript, we postulate that La^{3+} can alters voltage-sensing domain (VSD) activity through several possible mechanisms such as: (i) binding within the selectivity filter and allosterically modulating the VSDs, (ii) surface charge screening, or

(iii) a direct interaction of La^{3+} with the VSDs themselves. In Cav3.1 channels, the ionic current blockade by La^{3+} is generally attributed to binding near the selectivity filter (*e.g.* Mlinar and Enyeart, *J Physiol* 1993 PMID: 8271221, Beedle et al., *J Membr Biol* 2002 PMID: 12163980, Obejero-Paz et al., *JGP* 2004 PMID: 15572343), rather than to a direct interaction with the voltage sensor itself. Although the precise mechanism by which La^{3+} alters the voltage dependence of three out of four VSDs remains to be determined, the La^{3+} experiments resolve a longstanding conundrum in the field by showing that activation of VSDs precede pore opening, as in the other Cav channels.

We fully agree with the Reviewer that exploring Cav3.1 block by small-molecule pore blockers, like the Cav3 specific drug Z944 (Zhao et al., *Nature* 2019 PMID: 31766050), would provide valuable insights into the pharmacological mechanism of action. In this regard, our preliminary analysis of VSD behavior in the presence of Z944 revealed a perturbation pattern similar to that observed in the La^{3+} experiment, with VSD-III and VSD-IV being the most affected, while VSD-II remained unaltered. Interestingly, La^{3+} and Z944 perturbed VSDs in different manners, highlighting two specific mechanisms of pore blockade. These initial findings are part of an ongoing proposal, and a more detailed investigation is planned for future.

Together, these observations suggest that both La^{3+} (likely by binding near the selectivity filter) and Z944 (binding at the inner pore gate) can allosterically modulate VSD activation through distinct binding sites. Additionally, these findings indicate that pore blockers can influence VSD gating, challenging the long-standing assumption that pore blockades leave the function of VSDs untouched.

3-Building on the work of He et al. (2022, PMID: 35440630), who used structural and mutagenesis approaches to highlight the critical role of the positively charged Domain III S6cyto loop in low-voltage activation gating, this study employs VCF to demonstrate S6Cyto's influence on VSD activation (particularly VSD-IV). The authors propose that the positively charged S6Cyto electrostatically counteracts the VSD-IV deep resting state, thereby minimizing the energy required for activation and facilitating channel opening. However, direct mechanistic evidence supporting this model is lacking in the manuscript.

We do not fully agree with this comment. Our COVG-VCF experiments and the interpretation presented in the manuscript indeed are mechanistic. The VCF data presented in Figure 5 of the revised manuscript shows for the first time that $S6^{Cyto}$ stabilizes the resting state of VSD-IV and the associated model proposed is indeed the most parsimonious interpretation of the experimental data. Furthermore, the removal of the charges in $S6^{Cyto}$ also inhibited VSD-I and VSD-III, confirming that these two VSDs are indeed involved in the channel opening.

Ultimately, it may soon become possible to resolve the Cryo-EM structure of Cav3.1, including the $S6^{Cyto}$ region, possibly within nanodiscs and at hyperpolarized membrane

potentials. Such a structure would allow an even more direct mechanistic evaluation of the interactions between $S6^{Cyto}$ and the VSDs.

4-The authors suggest that "the CaV3.1 DII VSD is unlikely to contribute to pore opening". However, a study found that a spider toxin selectively targeting the DII VSD potently inhibits Cav3.1 current and impedes channel opening (PMID: 35858123), which indirectly suggests that the DII VSD is critical for pore opening.

We appreciate the Reviewer's comment and the opportunity to clarify this view. The paper referenced by the Reviewer (McArthur JR et al., *Elife* 2022 PMID: 35858123) dissects the effect of the spider venom peptide μ-Theraphotoxin Pn3a on T-type Ca^{2+} channels and demonstrates that Pn3a is highly selective for $Ca_V3.3$, but had minimal effect on $Ca_V3.1$ (the channel in our study) or $Ca_V3.2$. The authors also show that Pn3a interacts with the VSD-II paddle (S3–S4 region) of $Ca_V3.3$, which contains residues not conserved in $Ca_V3.1$. Thus, the study provides evidence for a functional role of the DII VSD in $Ca_V3.3$ gating but does not indicate that the $Ca_V3.1$ DII VSD contributes to pore opening, as $Ca_V3.1$ is largely insensitive to this toxin.

It would be of great interest to use COVG-VCF to explore the activity of the VSDs in the $Ca_V3.3$ with Pn3a to guide the design of more selective modulators that maintain isoform specificity.

In light of the Reviewer's comment, we have modified the Discussion (Page 8, lines 304-308) to include this reference.

"These results lend support to previous studies that concluded that the $Ca_V3.1$ Repeat-II is unlikely to contribute to pore opening [35], and stressed the preeminent role of VSD-I and -IV to voltage-dependent gating of $Ca_V3.1$ [36,37]. Interestingly, while VSD-II appears largely dispensable for gating in $Ca_V3.1$, it assumes a more prominent role in the opening of LVA $Ca_V3.3$ subtype, as reported by McArthur and colleagues [38]."

5-Overinterpretation of evolutionary claims：The assertion that "Nav-like VSDs emerged before sodium selectivity" is intriguing but remains speculative, and they provide no direct evidence for this sequence of events. The phylogenetic tree (Fig. 5a) lacks bootstrap values, weakening its robustness. Perform bootstrap analysis (1,000 replicates) to support the Cav3/Nav clade. In addition, the analysis includes only human channel sequences (19 total). This limited scope ignores potential insights from basal organisms (e.g., invertebrates or early vertebrates), which could provide stronger tests of evolutionary divergence. Without broader taxonomic representation, the tree may not accurately reflect deep evolutionary relationships.

Thank you for your question. Phylogenetic trees were constructed using the maximum likelihood approach and robustness of the nodes was assessed by bootstrap analysis (1,000 replicates). We have added bootstrapping values to the branches in Figure 6a and

Supplementary Figure 3b-c. Values obtained for the Ca_V3/Na_V clade ranged from 94 to 100, strongly supporting the vicinity of Na_V and Ca_V3 channels. Overall, the idea that Na_V and LVA Ca_V-channels have evolved from a common ancestor was first hypothesized by Hille nearly 30 years ago and our current work on the $Ca_V3.1$ VSDs, together with publicly available data, experimentally supports for the first time this long-standing hypothesis by looking at the VSDs properties of these channels. Importantly, comparison of the functional data of VSDs when overlayed upon this initial tree provides much commonality enhancing the view of the Hille 1984 hypothesis.

The presented phylogenetic tree and hypothesis is correctly placed within the discussion for some of the reasons illuminated by the Reviewers. We argue that the Hille (1984) hypothesis of the Na_V channel coming from a progenitor Ca_V channel is supported primarily by the functional data contained herein and secondarily supported by sequence analysis. It will be fascinating to obtain both Na_V and Ca_V channel recordings from “basal” organisms. However, the availability of clones and their expression does not have the broad coverage we have demonstrated within the current study.

We have modified the Discussion (Page 9, lines 330-335) as follows:

“We assembled the amino-acid sequences of all nineteen Ca_V- and Na_V-channels and subjected them to a preliminary phylogenetic analysis using a maximum-likelihood approach. Whether we used the full-length sequence (Supplementary Fig. 3b), the VSDs (Fig. 6a), or just the S4 regions (Supplementary Fig. 3c), a similar tree was produced: specifically, i) Na_V and LVA Ca_V channels shared a common ancestor as supported by the bootstrap values; and ii) LVA are more closely related to Na_V than to all other Ca_V channels (Fig. 6 and Supplementary Fig. 3).”

We have added tree-building method in Methods (Page 12, lines 460-466) as follows:

“Each separate alignment was utilized for maximum likelihood phylogenetic tree generation using the online program IQ-TREE (http://iqtree.cibiv.univie.ac.at). Output for each tree in Newick format was then tree assembled with the online program ETEToolkit [56] (http://etetoolkit.org/treeview/). All trees generated were qualitatively the same, node support was assessed via bootstrapping with 1,000 replicates. VSD (S1-S4) tree is shown in Fig. 6 and comparative trees of full-length sequence and the VSD S4 are shown in Supplementary Fig. 3b-c.”

6-According to the authors' phylogenetic tree analysis, Cav3 channels share a closer evolutionary relationship with Nav channel subtypes Nav1.8 and Nav1.9. However, Nav1.8 and Nav1.9 exhibit stark differences in activation kinetics: Nav1.9 opens near resting membrane potentials (low-threshold activation), similar to LVA Cav3 channels.Nav1.8 has the highest activation threshold among Nav channels, resembling

HVACav channels. How can this be reconciled?" The authors claim that Cav3.1 channels share high similarity in voltage-dependent properties with Nav1.4 and Nav1.5. However, Fig.5 shows that Nav1.4 VSDs activate at lower voltages than Cav3.1 VSDs. According to the authors' theory, Nav1.4 pore opening should therefore occur at lower voltages than Cav3.1. Yet in the figure, Nav1.4 pore opening occurs around -40 mV, while Cav3.1 opens near -60 mV. Why?

Thank you for raising this important point. By combining VCF functional data with phylogenetic trees based on sequence analysis of human channels, we concluded that Ca$_V$3 channels are more closely related to Na$_V$ channels than to HVA Ca$_V$ channels. Functionally, we found that the VSDs of Ca$_V$3.1 resemble those of Na$_V$1.4 and Na$_V$1.5 in that all of their VSDs are tuned to activate at hyperpolarized membrane potentials, near the resting potential of excitable cells (Fig. 6b in the revised manuscript). This feature has not been observed in any HVA CaV channels studied to date (Ca$_V$1.1, Ca$_V$1.2, Ca$_V$2.1, Ca$_V$2.2).

We currently do not know the precise voltage dependence of VSDs activation in Na$_V$1.8 and Na$_V$1.9, and the Reviewer's comment regarding their distinct voltage dependence of pore opening is valid. However, it is important to note that the voltage at which the pore opens depends not only on the voltage dependence of the VSDs but also on the energetic coupling strength between each VSD and the pore. For example, although the VSDs of Na$_V$1.4 activate at more hyperpolarized potentials than those of Ca$_V$3.1, it is reasonable to think that the Ca$_V$3.1 pore opens at lower voltages because of stronger coupling between VSD(s) and the pore (*i.e.* the active states of Ca$_V$3.1 VSDs may contribute more energy to stabilize the open state of the pore). Thus, channels with similar VSD voltage dependences may display different voltage dependences of pore opening.

Certainly, further functional studies and quantitative modeling of channel gating, will allow more direct comparisons between Ca$_V$3.1 VSDs and those of other channel subtypes, including Na$_V$1.8 and Na$_V$1.9.

-Specify the alignment algorithm and tree-building method (e.g., maximum-likelihood) in Methods.

Done. We have added the alignment algorithm and tree-building method in Methods (Page 12, lines 452-466) as follows:

"Reference human CaV and NaV channel protein sequences (UniProt accession no.: *CACNA1S*: Q13698, *CACNA1C*: Q13936-1, *CACNA1D*: Q01668-1, *CACNA1F*: O60840-1, *CACNA1A*: O00555-8, *CACNA1B*: Q00975-1, *CACNA1E*: Q15878-1, *CACNA1G*: O43497-2, *CACNA1H*: O95180-1, *CACNA1I*: Q9P0X4-1, *SCN1A*: P35498-1, *SCN2A*: Q99250-1, *SCN3A*: Q9NY46-1, *SCN4A*: P35499, *SCN5A*: Q14524-1, *SCN8A*: Q9UQD0-1, *SCN9A*: Q15858-1, *SCN10A*: Q9Y5Y9, *SCN11A*: Q9UI33-1) nineteen in all, were utilized for alignment with the program SeaView 5.0.5 [55]. Accession numbers and amino-

acids used for the alignment are listed in Supplementary Fig. 3a. Three separate sequence profiles were used: the full-length sequence; the individual VSD (S1-S4), and S4 sequences. Each separate alignment was utilized for maximum likelihood phylogenetic tree generation using the online program IQ-TREE (http://iqtree.cibiv.univie.ac.at). Output for each tree in Newick format was then tree assembled with the online program ETEToolkit [56] (http://etetoolkit.org/treeview/). All trees generated were qualitatively the same, node support was assessed via bootstrapping with 1,000 replicates. VSD (S1-S4) tree is shown in Fig. 6 and comparative trees of full-length sequence and the VSD S4 are shown in Supplementary Fig. 3b-c."

NCOMMS-25-53611-B RESPONSE TO REVIEWER COMMENTS

We sincerely thank the Reviewers for the positive appraisal of our revised version of the work. We have addressed all the remaining concerns and made changes to the revised manuscript accordingly.

Major changes in the manuscript are in blue color fonts.

Please find below our point-by-point answers to Reviewers' comments quoted in blue font, Italic.

Reviewer #1 (Remarks to the Author):

In the revised version of their manuscript, Angelini et al. have satisfied the great majority of my previous comments and criticisms and I consider the article suitable for publication.

Thank you! We have addressed all the concerns and made changes to the manuscript accordingly.

Most importantly, they have completely redrawn figure 3 and added the analysis of activation kinetics. This analysis demonstrates that activation of all four VSDs contains a component sufficiently fast to account for the rapid kinetics of pore opening. This additional data substantially improved the study. Of course, the observation that the bulk of the movement of VSDs I and IV (slow component) occurs after pore opening merits a brief explanatory statement. Do these VSDs have multiple activated states or is their partial S4 transition sufficient to activate pore opening?

Thank you for raising this point. VSD-I and VSD-IV display a slow component in the fluorescence signal. It is possible that these slow components reflect S4 transitions associated with slower voltage-dependent processes in channel gating (*e.g.* inactivation).

Regarding the relative proportion between fast and slow components, it is not a reflection of the extent of the VSD movement, but rather of the extent of the quenching process experienced by the fluorophore. The relative amplitude of the two components in the fluorescence signal does not necessarily correlate with the magnitude of the VSD movement: *e.g.* a large conformational change may be associated with a low probability of fluorophore quenching/unquenching, generating a small ΔF. On the other hand, a subtle conformational change that causes a high probability of quenching/unquenching of the fluorophore will generate a large fluorescence signal.

As suggested by the Reviewer, we have included the following sentence in the Results:

"The fast components of VSD-I (at −20mV, τ_{fast} = 2.6 ± 0.26 ms, accounting for 28% ± 3% of total amplitude) and VSD-IV (τ_{fast} = 2.3 ± 0.23 ms, 47% ± 7% of total amplitude) were

also comparable to the rate of channel opening (Fig. 3b, e, f). Both VSDs also displayed a slower fluorescence component. Although we did not further investigate the origin of these signals, they may report slower conformational changes of the protein associated with channel gating other than activation (*e.g.* inactivation).”

The authors also have removed the rather misleading reference to evolution from the title. However other than this, they chose to adhere to their interpretation of the data in terms of channel evolution. While their sequence analysis adds valuable new evidence to this discussion, the contribution of their functional analysis to this debate is questionable. Considering the existing VCF data shown in figure 6, the statement, “By comparing the VSDs of CaV1 12-14,17, CaV2 15,16, CaV3 (this work) and NaV channels 49,50 (Fig. 6), we provide evidence that the CaV3.1 VSDs operate more similarly to those of a NaV channels than HVA CaV-channels, in terms of overall voltage sensitivity and range of half-activation potentials (Fig. 6).” lacks sufficient experimental support. The authors compare 1 of 3 CaV3 channels with 2 of 10 NaV channels. Indeed, the narrow range of activation voltages of CaV3.1 is similar to that of NaV1.4; however, its voltage sensitivity or voltage-dependence of activation are not at all similar. Furthermore, the narrow range of CaV3.1 VSD activation is dissimilar to that of the other example NaV1.5 (the activation voltages of which ranges over 50 mV and thus is much more similar to that of CaV1 channels). At this point, we do not know the properties of the other two CaV3 channels, neither do we know whether the other eight NaV channels exhibit properties more like NaV1.4 or NaV1.5, or show even greater differences to those of CaV3.1. Consequently, the current study adds one important data point to this question, but is far from demonstrating that, “... our VCF data support experimentally the CaV3.1 evolutionary vicinity to NaV-channels and their distinction from HVA CaV channels.”

As requested, we have toned down our Discussion and removed the following sentence: “our VCF data support experimentally the CaV3.1 evolutionary vicinity to NaV channels and their distinction from HVA CaV channels.”

We have revised the Discussion as follows:

“The properties of VSDs provide a further means to explore the evolution of these channels.

The VSDs of CaV3.1 have a noteworthy similarity with the VSDs of NaV-channels studied by VCF to date (NaV1.4 [49] and NaV1.5 [50]): they all display hyperpolarized activation V_{half} (Fig. 6). By contrast, the VSD properties of HVA CaV-channels resolved so far (CaV1.1 [14,17], CaV1.2 [12,13,] CaV2.1 [16] and CaV2.2 [15]) activate at a broader range of V_{half} (Fig. 6b). While this observation is yet tentative, pending the characterization of all CaV and NaV VSDs, it prompted us to examine the evolutionary hypothesis of CaV and NaV channels, in light of the current theory of NaV evolving from CaV [47,51-53.] We assembled the amino-acid sequences of all nineteen CaV- and NaV-channels and subjected them to a

preliminary phylogenetic analysis using a maximum-likelihood approach. Whether we used the full-length sequence (Supplementary Fig. 3b), the VSDs (Fig. 6a), or just the S4 regions (Supplementary Fig. 3c), a similar tree was produced: specifically, i) Nav and LVA Cav channels shared a common ancestor as supported by the bootstrap values; and ii) LVA are more closely related to Nav than to all other Cav channels (Fig. 6 and Supplementary Fig. 3).

Furthermore, the functional similarity with regards to LVA Cav and Nav in controlling or regulating neuronal excitability presents corroborating evidence for evolutionary relatedness. The overlap of some aspects of VSD functional properties and sequence analyses (Fig. 6) agree with the theory that LVA Cav and Nav share a common ancestor, as proposed 20 years ago [47]."

Having said this, I want to re-emphasize the importance and excellent quality of this VCF study and that I am looking forward to seeing it published in Nature Communications.

Thank you for the constructive feedback.

Reviewer #2 (Remarks to the Author):

I co-reviewed this manuscript with one of the reviewers who provided the listed reports. This is part of the Nature Communications initiative to facilitate training in peer review and to provide appropriate recognition for Early Career Researchers who co-review manuscripts.

Thank you for co-reviewing our manuscript.

Reviewer #3 (Remarks to the Author):

Thank you for your detailed responses to the previous comments.

Thank you for your constructive feedback.

Regarding point 2, the authors state that the Cav3 channel-specific inhibitor Z944 was used. However, no corresponding data is presented in the response to support this crucial methodological claim.

In our previous response to Reviewer's comments, we mentioned ongoing experiments with the Z944 inhibitor. Although these data are not included in the present study, we are happy to share these preliminary results with the Reviewer.

[REDACTED]

[REDACTED]

[REDACTED]

[REDACTED]

[REDACTED]

[REDACTED]

[REDACTED]

[REDACTED]
